# SWI/SNF-Like Chromatin Remodeling Factor Fun30 Supports Point Centromere Function in *S. cerevisiae*

**DOI:** 10.1371/journal.pgen.1002974

**Published:** 2012-09-27

**Authors:** Mickaël Durand-Dubief, William Ryan Will, Edoardo Petrini, Delphine Theodorou, Rachael R. Harris, Margaret R. Crawford, Konrad Paszkiewicz, Felix Krueger, Rosa Maria Correra, Anna T. Vetter, J. Ross Miller, Nicholas A. Kent, Patrick Varga-Weisz

**Affiliations:** 1 Nuclear Dynamics, Babraham Institute, Cambridge, United Kingdom; 2 Cardiff School of Biosciences, Cardiff University, Cardiff, United Kingdom; 3 School of Biosciences, University of Exeter, Exeter, United Kingdom; 4 Bioinformatics, Babraham Institute, Cambridge, United Kingdom; Duke University, United States of America

## Abstract

Budding yeast centromeres are sequence-defined point centromeres and are, unlike in many other organisms, not embedded in heterochromatin. Here we show that Fun30, a poorly understood SWI/SNF-like chromatin remodeling factor conserved in humans, promotes point centromere function through the formation of correct chromatin architecture at centromeres. Our determination of the genome-wide binding and nucleosome positioning properties of Fun30 shows that this enzyme is consistently enriched over centromeres and that a majority of *CENs* show Fun30-dependent changes in flanking nucleosome position and/or *CEN* core micrococcal nuclease accessibility. Fun30 deletion leads to defects in histone variant Htz1 occupancy genome-wide, including at and around most centromeres. *FUN30* genetically interacts with *CSE4*, coding for the centromere-specific variant of histone H3, and counteracts the detrimental effect of transcription through centromeres on chromosome segregation and suppresses transcriptional noise over centromere *CEN3*. Previous work has shown a requirement for fission yeast and mammalian homologs of Fun30 in heterochromatin assembly. As centromeres in budding yeast are not embedded in heterochromatin, our findings indicate a direct role of Fun30 in centromere chromatin by promoting correct chromatin architecture.

## Author Summary

Centromeres are essential to chromatin structures, providing a binding platform for the mitotic spindle. Defects in centromere structure or function can lead to chromosome missegregation or chromosome breakage. This, in turn, can cause cancer in metazoans. Centromeres are defined by specialized chromatin that contains the histone H3 variant CENP-A (also called CenH3, or Cse4 in budding yeast), and transcription over centromeres is tightly controlled. Budding yeast centromeres are composed of a single nucleosome containing the essential Cse4. Loss of one of these specialized centromeric nucleosomes can lead to chromosome missegregation during mitosis followed by cell death. We provide evidence that energy-dependent chromatin remodeling factor Fun30 supports faithful chromosome segregation, especially when centromere structure is challenged by mutation of Cse4 or by forced transcription through centromeres, which disrupt centromere structure. We show that Fun30 binds to centromeres and that loss of Fun30 leads to various defects in centromere chromatin, suggesting a direct role for Fun30 in promoting normal centromere function. Our analysis shows that Fun30 affects nucleosome positioning at many genomic sites, including centromeres, and is required for normal occupancy of histone variant Htz1. In the absence of Fun30, we detect an increase in transcription through centromeres. We suggest that an important function of Fun30 is to limit transcription over centromeres.

## Introduction

The functional state of chromatin domains results from the action of multiple determinants, including histone modifications, histone variants, nonhistone proteins and nucleosome remodeling factors. The inclusion of specific histone variants is essential for the organisation of chromatin to delineate specific domains [1]. For example, histone H3 variant CENP-A (CENH3) and its orthologs characterize centromeric regions [2]. The histone H2A variant H2AZ (Htz1 in budding yeast) demarcates many promoters and boundary elements in yeast and other organisms [3]. The distribution of histones and specific histone variants, in turn, is regulated by SWI/SNF2-like ATP-dependent remodeling activities. The Fun30/SMARCAD1/Etl1 family is a poorly characterized class of SWI/SNF-like factors [4]. In mice SMARCAD1 (also referred to as ETL1) is important for normal development [5] and is implicated in pluripotency and self renewal in embryonic stem cells [6]. SMARCAD1 has a role in maintenance of silent chromatin through replication in mammalian cells [7]. In *Saccharomyces cerevisiae* the unique SMARCAD1 homolog Fun30 is required for silencing in heterochromatin loci [8], [9]. Fun30 has nucleosome remodeling activity *in vitro* and affects chromatin structure *in vivo* [8]–[10]. Fission yeast has three genes coding for Fun30-like factors, one of which, FFT3, has been shown to function at boundary elements, protecting heterochromatin from euchromatin invasion [11].

The analysis of centromere establishment and maintenance has provided many important insights into how various chromatin factors cooperate to assemble a very specific and essential chromatin configuration. Centromeres serve as attachment anchors for kinetochore proteins, which, in turn, interact with microtubules of the mitotic spindle (reviewed in: [12], [13]). A hallmark of all eukaryotic centromeres is the centromeric histone H3 variant CENP-A (termed Cse4 in budding yeast) that provides an essential platform for kinetochore assembly and subsequent chromosome segregation [13], [14]. In budding yeast centromeres are well defined and composed of a single Cse4-containing variant nucleosome for each chromosome, each occupying approximately 125 bases pairs comprising three regions (CDE I, CDE II, CDE III) [13], [15]–[21]. Multiple mechanisms contribute to the specific localization of Cse4, including Scm3, a Cse4-specific histone chaperone [22], [23], and the regulated Cse4 removal and degradation at extra-centromeric sites [24]–[26]. Each single point centromere is essential for viability. Therefore, these point centromere nucleosomes provide a unique model system to explore how specific chromatin configurations are assembled and maintained.

To gain insight into Fun30 function, we mapped Fun30 binding sites genome-wide and found that it bound chromatin at specific loci, particularly at the centromeres. We found that Fun30 supports chromosome segregation, and determines nucleosome positioning at many sites, including the majority of centromeres. Fun30 has a major impact on Htz1 occupancy genome-wide, including around centromeres. We propose that Fun30 assists faithful chromosome segregation by promoting a correct chromatin infrastructure at and around centromeres and limits perturbation of centromeres through transcription.

## Results

### Fun30 Is Recruited to Specific Genomic Loci including Centromeres

**Figure 1:**
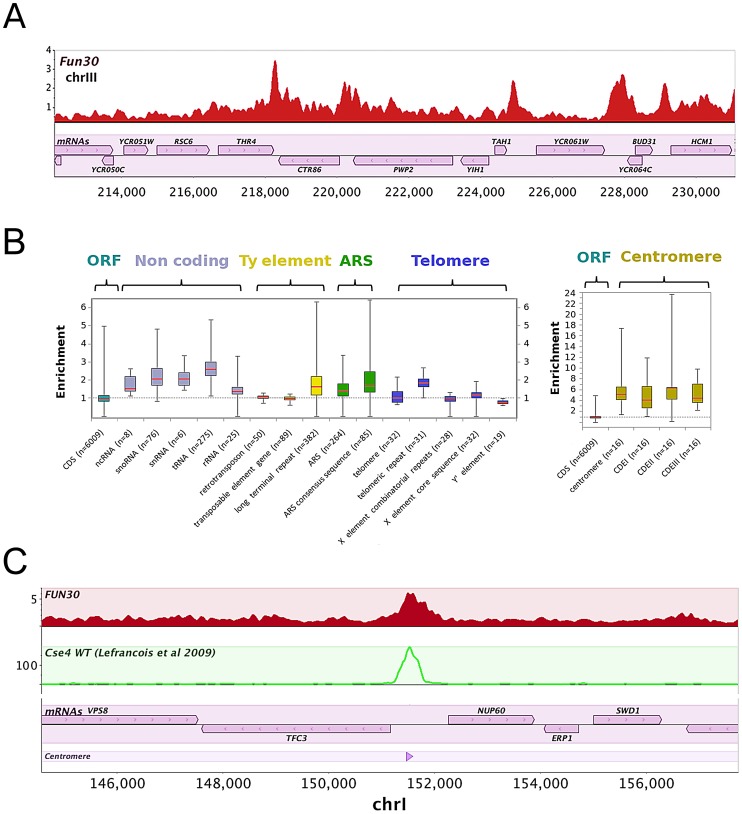
Fun30 is localized to specific regions in the genome, including centromeres. A) Overview of ChIP-Seq binding profile of Fun30 for a segment of chromosome III. The lower track of the figure shows open reading frames (ORFs) and their orientations. B) Box plot analysis of Fun30 enrichment over different genomic features. The lines inside the boxes represent the 50^th^ percentile (the median) values. The bottom and top of the boxes represent respectively the 25^th^ and 75^th^ percentile (the lower and upper quartiles, respectively). The ends of the whiskers represent minimum and maximum values. C) Binding profile of Fun30 in a selected region of chromosome I including the centromere. The second track shows the binding profile of Cse4 (ChIP-Seq data extracted from [86]). The bottom tracks show open reading frames (ORFs) and their orientations and the position of centromere *CEN1*.

To obtain insights into the biological role of Fun30, we performed chromatin immunoprecipitation followed by high-throughput sequencing (ChIP-Seq) to obtain a genome-wide binding profile for Fun30. We obtained more than 4.8×10^+6^ sequence reads - *i.e.* 14-fold coverage of the genome - giving us a comprehensive insight into Fun30 enrichment across the chromatin. A browser overview showed that peaks of Fun30 enrichment are found predominantly at intergenic regions (Figure 1A). Fun30 is relatively depleted within ORFs compared to intergenic sites and Fun30 peaks are predominantly over the 3′ end of genes compared to the 5′ start site (Figure 1B, Figure S1).

To further explore the binding of Fun30 to intergenic sites, we analyzed its association with genomic features (Figure 1B). Several genomic elements show preferential enrichment of Fun30 including tRNAs genes, small nuclear RNA (snRNA) genes, small nucleolar RNA (snoRNAs) genes, Long Terminal Repeats and Autonomous Replicating Sequence regions (ARSs) (Figure 1B). We also noticed telomeric repeats are enriched in Fun30 whereas subtelomeric elements X and Y′ are not (Figure 1B, left panel). Interestingly centromeric regions show the greatest enrichment of Fun30 compared to other sites (Figure 1B, right panel, Figure 1C). The enrichment of Fun30 at centromeres was confirmed by chromatin immunoprecipitation experiments followed by quantification using PCR (Figure S2).

### *FUN30* Deletion Leads to Upregulation of Genes Involved in Chromosome Segregation

**Figure 2:**
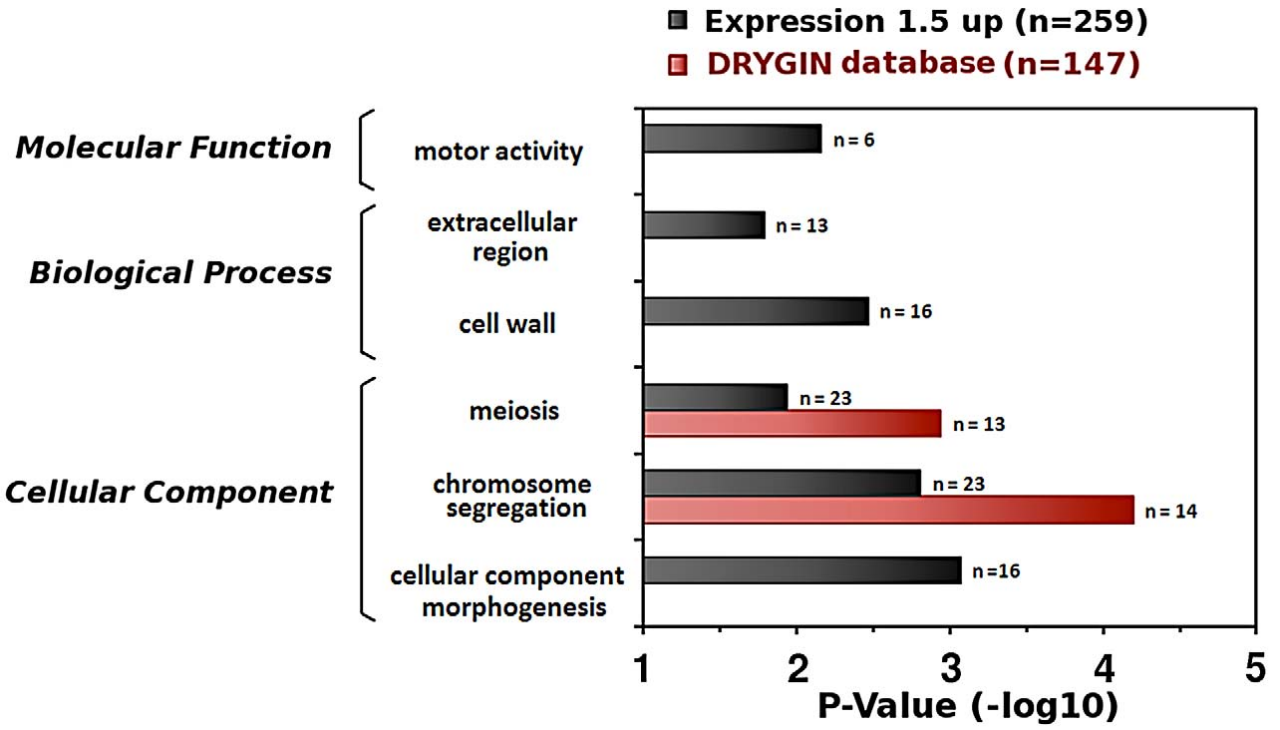
Gene expression profile and genetic interactions indicate roles for Fun30 in meiosis and chromosome segregation. Merged histogram of Gene Ontology analysis for 1.5-fold up-regulated mRNAs (n = 259 genes with annotated function in GO) in *Δfun30* cells compared to wildtype cells and for genes interacting synthetically with *FUN30* from the DRYGIN database (n = 147, Epsilon cutoff 0.04 and P-value cutoff 0.05) [37]. Identified gene categories are indicated. Abscises show the P value (−log 10). The gene list is in supplementary data.

To further understand how Fun30 might have a role in chromosome segregation and to test direct versus indirect roles, we performed expression profiling of mRNA using RNA-seq in *Δfun30* deletion and control (‘wildtype’) cells. A ‘global expression profile’ analysis [27] indicated that Fun30 activity is largely required to silence genes (Figure S3). We employed a 1.5-fold cutoff value to define lists of *FUN30*-deletion affected genes, identifying 255 genes which were downregulated and 573 genes which were upregulated (Table S1). To investigate if genes involved in specific cellular processes were affected by *FUN30* deletion, the up or down-regulated genes were submitted to Gene Ontology (GO) analysis [28] by applying a p-value cutoff (*P*<0.05). This analysis did not revealed significant GO terms in the group of downregulated genes. The upregulated gene group showed several genes involved in chromosome segregation (*pvalue^−log10^* = 2.8) and meiosis (*pvalue^−log10^* = 1.9) (Figure 2). The deletion of *FUN30* caused the upregulation of genes belonging to the anaphase promoting complex (AMA1, APC1, APC2, APC4, APC2, APC9, CDC26) which is required for sister chromatid separation and exit from mitosis [29]. Other upregulated genes are components of the kinetochore or involved in its assembly, such as IML3/MCM19 [30], [31]), CNN1 [32], [33]), DAM1 [34], [35]), TID3 [36]). The hypergeometric distribution analysis revealed only a poor relationship between Fun30 recruitment and upregulated (*P* = 0.042) or downregulated genes (*P* = 0.012). Thus, the upregulation of genes involved in these specific pathways appears to be a cellular response to the absence of Fun30 function. To explore this further, we investigated a quantitative genetic interaction profile database containing 75% of all genes in *S. cerevisiae* [37] (http://drygin.ccbr.utoronto.ca/) and found 147 genes (SGA 0.04, P<0.05 cutoff) that have a significantly similar genetic interaction profile as Fun30. Interestingly analysis of this list of genes by Gene Ontology also reveals roles in meiosis and chromosome segregation (Figure 2). This analysis shows significant negative genetic interactions with several genes involved in the spindle checkpoint (MAD3, BUB1, BUB3) (p-values: 3.01×10^−18^, 1.27×10^−3^ and 2×10^−4^) and kinetochore formation (NDC10, AME1) (p-values: 8.45×10^−18^ and 6.25×10^−3^) [38], [39]. The analysis also indicates a genetic similarity to components of the TRAMP complex, which, in turn, has been linked to chromosome segregation [40]. Together, these results suggest a role of Fun30 in chromosome segregation.

### *FUN30* Genetically Interacts with *CSE4*

**Figure 3:**
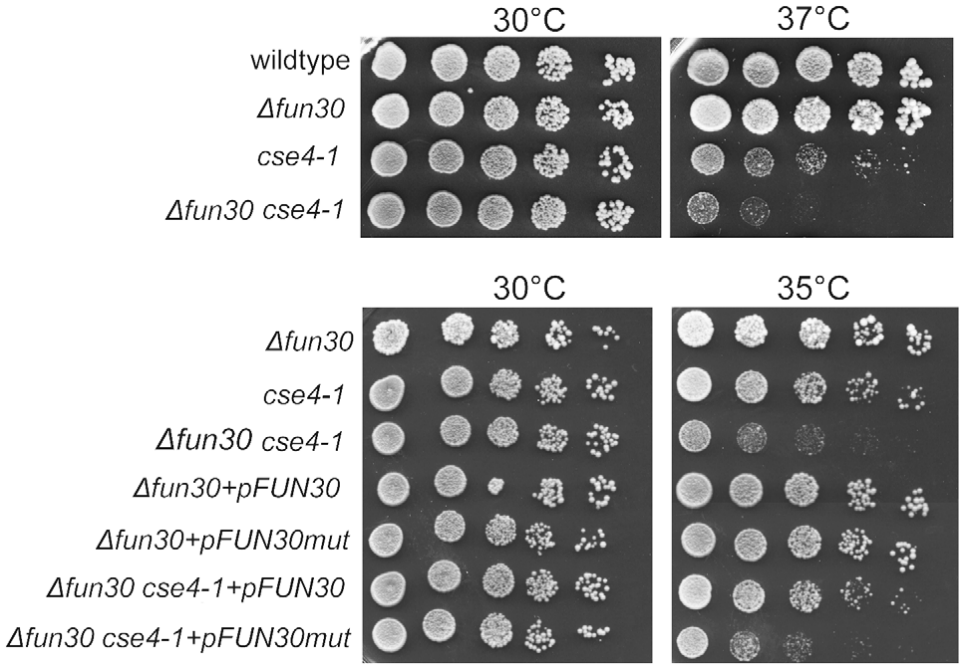
Fun30 is required when Cse4 function is compromised. Growth of the double mutant *Δfun30 cse4-1* is strongly affected at semi-restrictive temperatures. Fivefold dilutions of wildtype (BY4741/Y00000), *Δfun30* (Y00389), *cse4-1* (AHY666) and *Δfun30 cse4-1* (SC53) cells were plated onto YPD plates and incubated at indicated temperatures for 3 days. Lower panels: Fun30 activity is restored by expressing wildtype Fun30 *in trans*, but not Fun30 with a point mutation in the ATPase domain; Cells were spotted on media with 2% glucose and grown for 3 days at 30°C or 35°C.

Deletion of Fun30 alone does not significantly affect viability, whereas overexpression of Fun30 results in chromosome segregation defects [41]. To explore the role of Fun30 in centromere function, we used the conditional *cse4-1^ts^* mutant with an amino acid substitution [42] which leads to reduced Ctf3, Ctf19, Ndc10 and Scm3 binding over the centromere at the nonpermissive temperature (38°C) and causes cell cycle arrest in G2 phase accompanied by short bipolar mitotic spindles at 38°C [23], [42], [43]. At the permissive temperature all strains grew well (Figure 3, left panels, 30°C). At a semi-permissive temperature, control (wildtype) and *Δfun30* single mutant cells did not show growth defects, but growth of the *cse4-1* mutant was reduced, as expected (Figure 3, right panels, 37°C, 35°C). However, the double mutant *Δfun30 cse4-1* was significantly more inhibited (Figure 3, right panels, 37°C, 35°C). Growth could be rescued by expression of wildtype Fun30 *in trans*, but not by an ATP-binding site mutant Fun30 (Figure 3, lower right panel). These results therefore suggest a link between chromatin remodeling by Fun30 to centromeric function.

### Fun30 Counteracts Segregation Defects Mediated by Transcription through Centromeres

**Figure 4:**
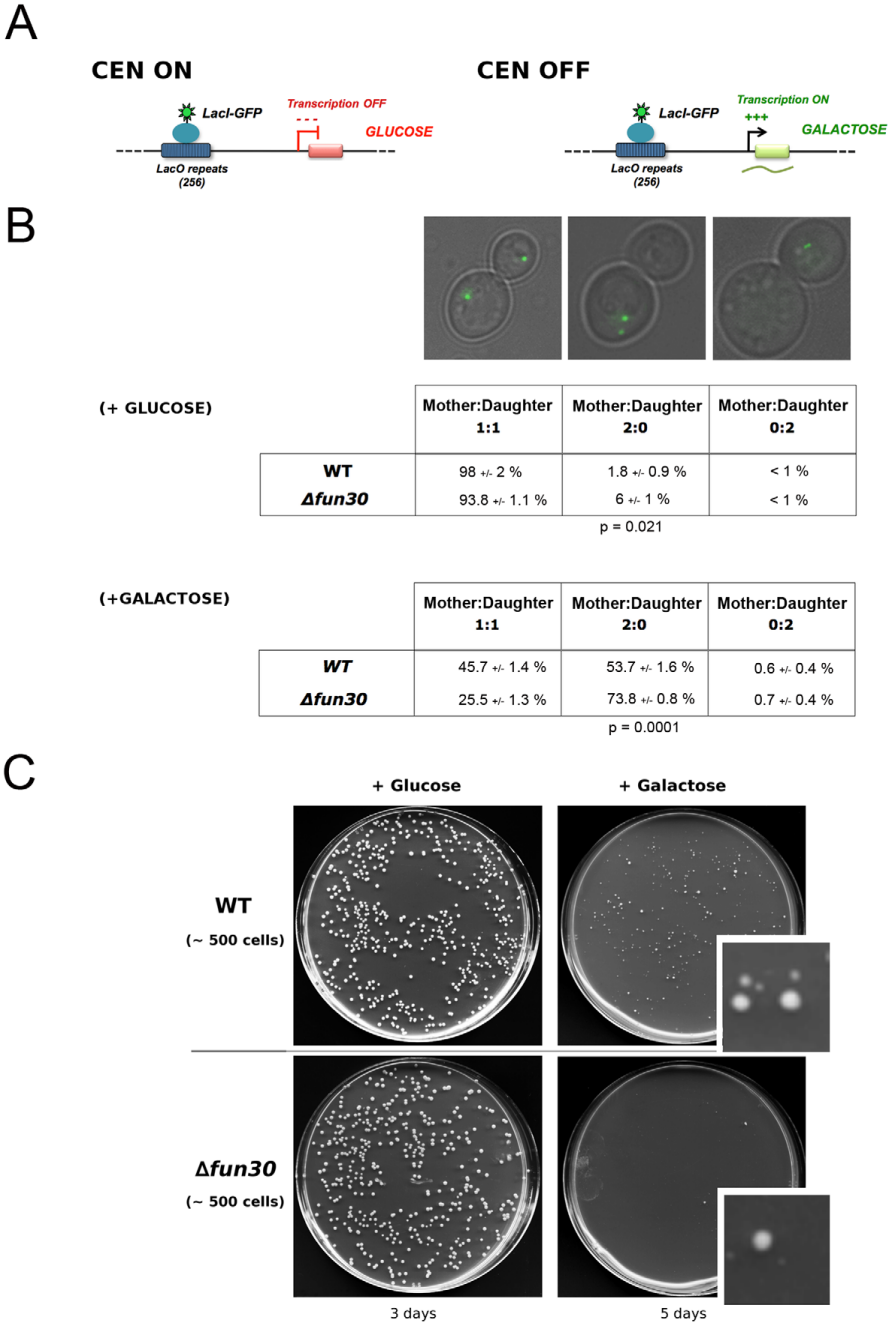
Absence of Fun30 increases chromosome segregation defects upon transcription through a centromere. A) Schematic depiction of the transcription-regulated centromere system. In the presence of glucose transcription through centromere III is repressed and the centromere functions normally (left panel). Galactose induces transcription that perturbs centromere function (right panel). Chromosome segregation is visualized by following the fluorescent dot, due to binding of LacI-GFP to a LacO array proximal to the centromere [45]. B) Chromosome segregation defects/delays in cells with a functional *CENIII* (+ glucose) or a transcription-disrupted *CENIII* (+ galactose) in the presence and absence of Fun30. Three categories of cells were scored: GFP dots separated with one in the mother cell and one in the daughter cell (bud, column 1), two dots in the mother cell (column 2), two dots in the daughter cell. Values represent the mean percentage of cells −/+ standard deviation of three experiments in which each time 200 cells were counted; the p values are derived from t-tests for the two dots in mother cases. C) ∼500 cells with or without Fun30, containing the conditional centromere were plated on glucose and incubated 3 days or on galactose and incubated 5 days. On glucose there were about 500 colonies irrespective of the presence or absence of Fun30, with less than 5% of the colonies being small. When grown in the presence of galactose, only ∼250 of the control cells grew to colonies, with ∼40 being large and the remainder small colonies. The *fun30-*deleted cells showed only 2 large colonies in this condition and ∼90 tiny colonies (see inserts). Repeat experiments gave similar results.

Inhibition of transcription through centromeres is required for the *de novo* establishment and maintenance of centromere function [44]–[46]. Consequently, forcing transcription through a centromere disrupts its normal function [47]–[49]. To test if Fun30 has a role in chromosome segregation when centromere function is perturbed, we employed a yeast strain where transcription through *CEN3* can be induced from a centromere proximal *GAL1* promoter by addition of galactose, and segregation can be monitored using live cell marking of chromosome III [45] (Figure 4A). Deletion of *FUN30* did not have a noticeable effect on transcription driven from the *GAL1* promoter at the *CEN3* (Figure S4). We examined chromosome segregation by determining if the GFP dots, marking chromosome III, are segregated into both mother and daughter cells, or remain in the mother cell or are both found in the daughter cell. In the absence of transcription through *CEN3*, ∼1% of control cells showed some segregation defect. Deletion of *FUN*30 increased the number of cells with both copies of chromosome III remaining in the mother cell ∼3 fold, indicative of a segregation defect or delay (Figure 4B). When transcription was induced, segregation defects increased dramatically in the control cells and this was further increased when *FUN30* was deleted (Figure 4B). Importantly, persistent transcription over days led to a substantial loss of viability when *FUN30* was deleted (Figure 4C). Therefore, it is possible that Fun30 affects events downstream of the transcription process, *e.g.*, re-establishment of centromeric and pericentromeric chromatin.

**Figure 5:**
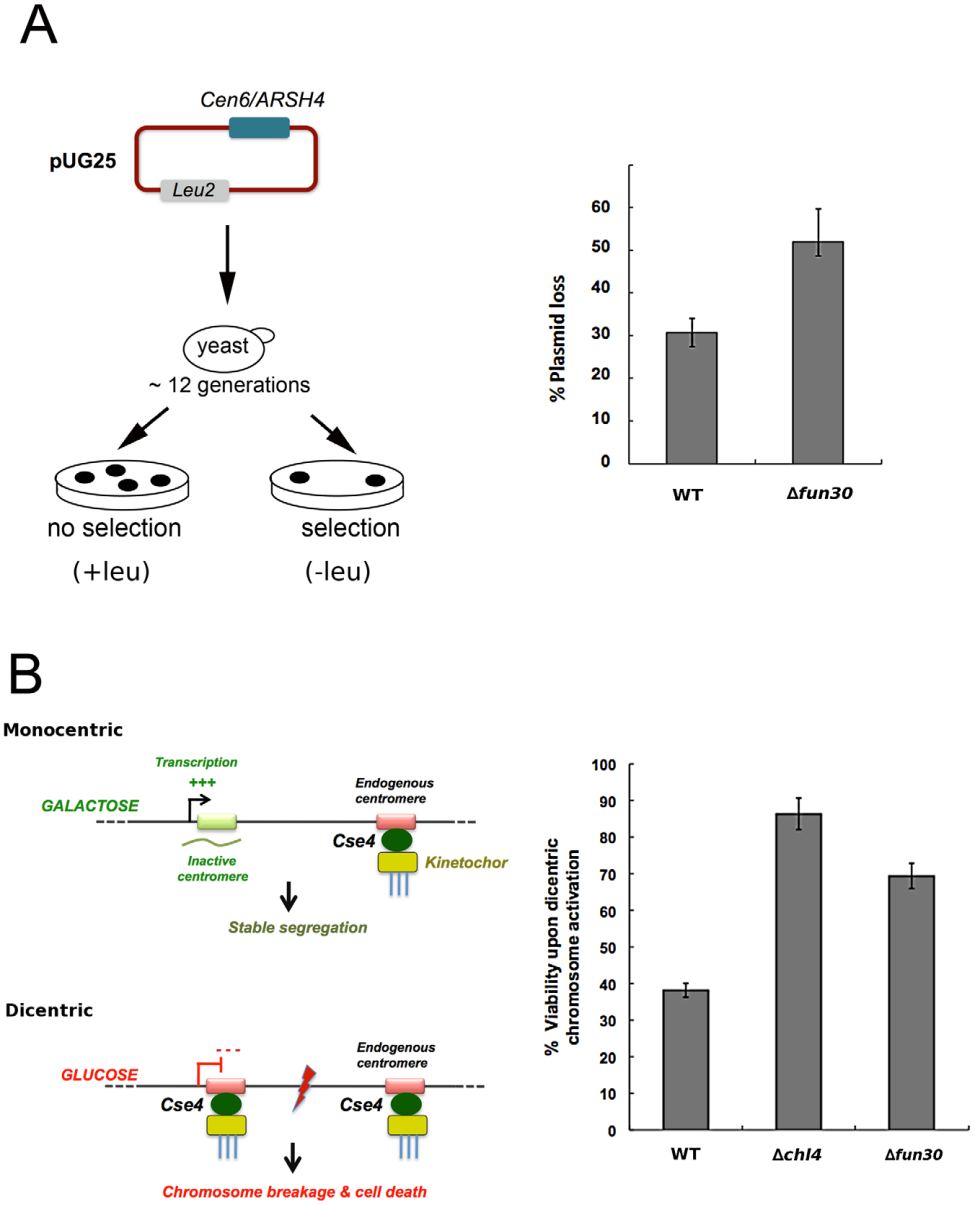
*FUN30* deletion counteracts viability defects upon formation of a dicentric chromosome. A) *Δfun30* cells show increased rates of loss of a circular minichromosome (pUG25), the left diagram illustrates the assay, the right panel shows % of plasmid loss in wildtype (wt) and f*un30-*deleted cells, shown is the average of two experiments, bars represent minimum and maximum values. B) Left panel: Diagram illustrating the dicentric chromosome breakage assay [46]. In the presence of galactose, the ectopic formation of a second centromere on chromosome III is suppressed through transcription of the locus. In the presence of glucose, the suppression of transcription allows formation of a second centromere on the same chromosome, which ultimately leads to chromosome breakage and loss of viability. Right panel: Deletion of *FUN30* promotes viability on induction of a dicentric chromosome, to a comparable extent as centromere establishment factor *CHL4*. More than 500 colonies/plate were counted for cells grown in galactose and the corresponding number of colonies were established for cells grown in glucose. Shown is a representative experiment, error bars represent 10% confidence interval.

A minichromosome loss assay with a plasmid bearing the centromere sequence of chromosome VI showed that *FUN30* was required for maintenance of this chromosome through multiple cell generations (Figure 5A). Defects in various pathways, including DNA replication, could explain this. However, given the centromeric localization of Fun30, one plausible explanation is that Fun30 is involved in the formation of a functional centromere *de novo* on naked centromere DNA. To explore this further, we employed a conditional galactose-regulated dicentric chromosome and the fact that multiple centromeres are deleterious in yeast [46] (Figure 5B, left panel). Activation of the second centromere by suppression of transcription through it results in chromosome breakage and loss of viability [46]. Mutations affecting centromere establishment, such as deletion of *CHL4*, an outer kinetochore component, result in a effective suppression of this dicentric chromosome breakage [46]. We found that deletion of *FUN30* promoted cell viability to almost the same extent as deletion of *CHL4* (Figure 5B, right panel), suggesting that Fun30 might assist activation of a functional dicentric chromosome, and, therefore, the establishment of a centromere *de novo*.

**Figure 6:**
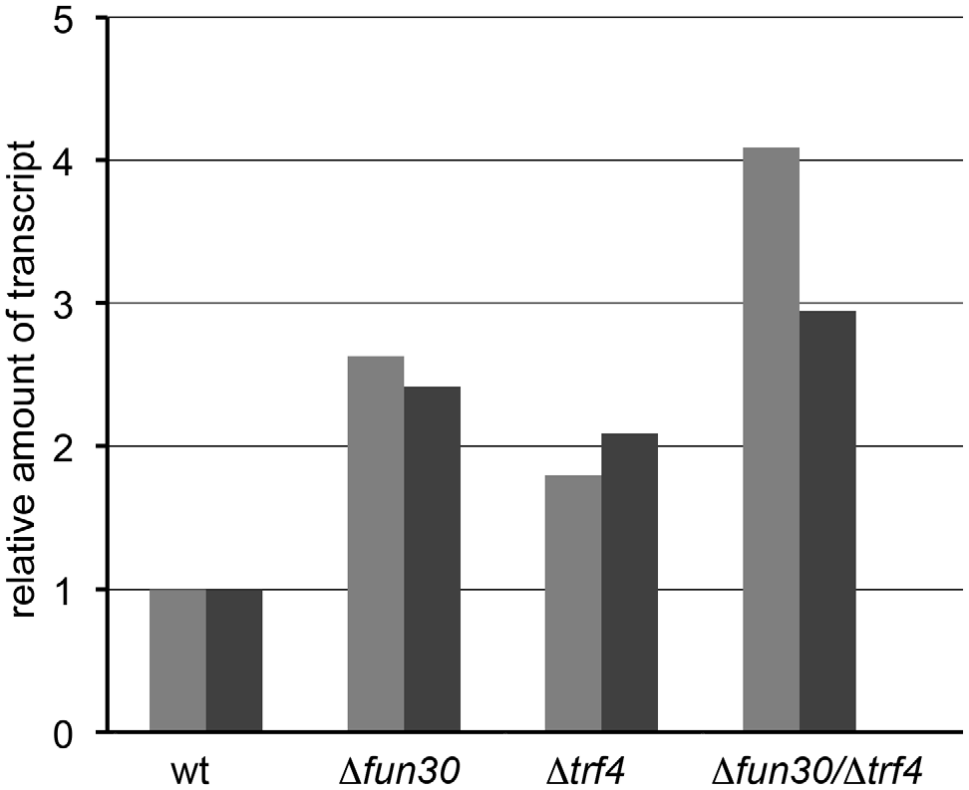
Loss of Fun30 leads to increased Transcription at centromere regions. Analysis of transcript levels at *CEN3* region by RT-qPCR in wildtype yeast (wt), the corresponding *Δfun30* mutant, *Δtrf4* mutant and the double mutant *Δfun30 Δtrf4* strains. Primers PM22/48 detecting transcripts directly over *CEN3* were used to amplify cDNA. The graph reports the relative amount of transcript compared to a control gene that is not regulated by Fun30. Similar results were obtained when we examined absolute amounts.

Because Fun30 has been linked to gene silencing, we asked if it might be required to silence transcription within the centromeres. We tested how centromere silencing was affected in the *Δfun30* mutant by measuring transcript levels for the *CEN3* region where a cryptic unstable transcript has been detected upon deletion of *PAP2*, the gene for Trf4, a component of the TRAMP complex involved in RNA surveillance and noncoding RNA degradation [50]–[53]. We found that deletion of *FUN30* increased the amount of transcript over the centromere compared to control to the same amount as seen when *TRF4* was deleted (Figure 6, wt, *Δfun30*, *Δtrf4*). Double deletion of *FUN30* and *TRF4* increased the amount of detectable transcript even further (Figure 6, *Δfun30 Δtrf4*).

Together, our data suggest that Fun30 promotes faithful chromosome segregation when centromere structure is challenged and this may be linked to Fun30's role in gene silencing.

### Fun30 Is Required for Normal *CEN*-Flanking Nucleosome Positioning and/or *CEN* Core Particle Structure

Next, we tried to elucidate if Fun30 promotes chromosome segregation by ensuring a correct centromere chromatin structure, in line with its binding to centromeres.

**Figure 7:**
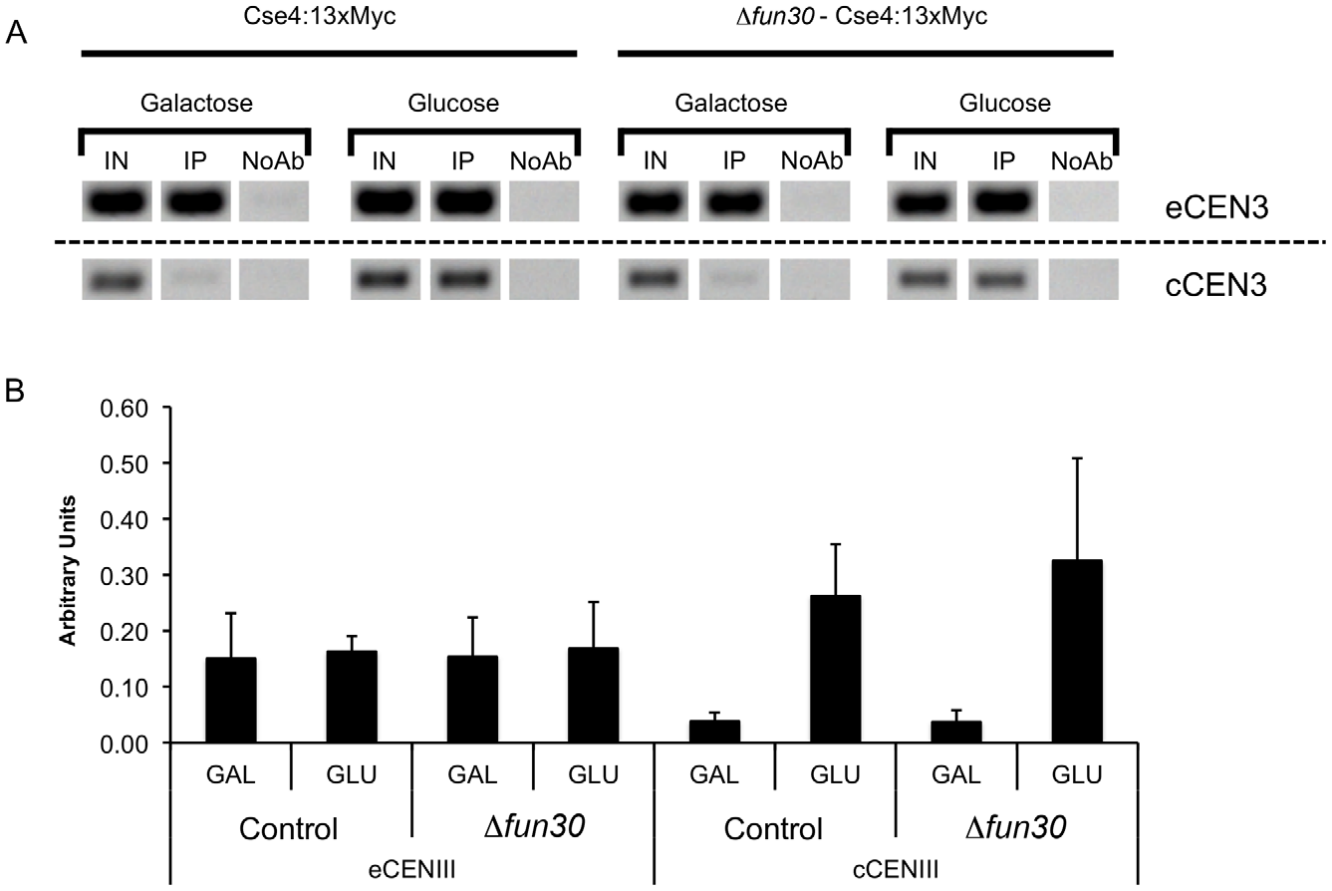
Deletion of *FUN30* does not affect Cse4 occupancy at a constitutive and an induced centromere. A) Cse4 histone variant occupancy at the endogenous *CEN3* locus (eCEN3) and at a conditional *CEN3* locus (cCEN3) when it is repressed (in the presence of galactose) or induced (glucose) by ChIP. Control (SC138) and *Δfun30* (SC140) cells are grown in YP Gal until≈1 OD, then shifted in YP Glu or YP Gal containing 15 µg/ml of nocodazole and incubated for 4 hours. Cse4-Myc associated chromatin was immunoprecipitated and the immunoprecipitated DNA was analyzed by PCR followed by agarose gel electrophorsis and ethidium bromide staining to visualize the DNA fragments. PCR was with the primers described in Table 3. Shown are the relevant, cropped out bands from a single, representative gel, input was 1/243th of the immunoprecipitated material. B) A bar plot reporting the enrichment over the input, expressed in arbitrary units, of three independent experiments as shown in (A). Quantitation of bands was carried out using ImageJ software, error bars are standard deviation from the average.

Our genome-wide analysis of Fun30 binding and the impact of *FUN30* deletion on histone H3 occupancy indicates that Fun30 binds preferentially at the terminator region of genes and that its binding there is linked to a loss of histone H3 occupancy at this region (Figures S1, S5). Thus, our data indicate that Fun30 is involved in nucleosome removal in intergenic regions and suggest that Fun30 may promote occupancy of the centromeric Cse4 containing nucleosome by favoring removal of canonical nucleosomes over the centromere. To test this idea, we measured Cse4 occupancy over the endogenous, constitutive *CEN3* centromere and over an induced centromere in control and *fun30* deleted cells. This analysis did not show significant changes in Cse4 occupancy upon *fun30*-deletion (Figure 7).

**Figure 8:**
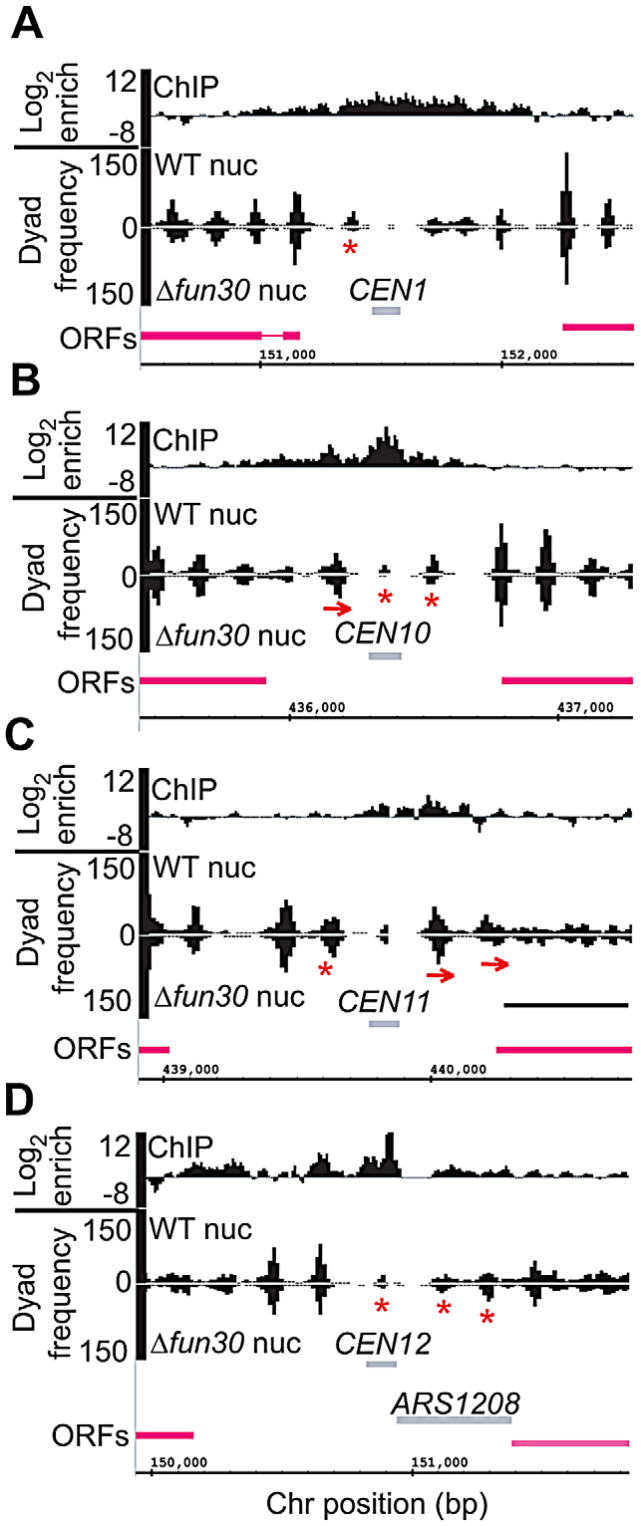
Fun30 is required for normal *CEN*-flanking nucleosome positioning and/or *CEN* core particle structure. A) Genome browser trace of Fun30 ChIP enrichment and nucleosome dyad frequency centred on and surrounding yeast *CEN1*. The upper trace shows Log_2_ Fun30 ChIP-seq enrichment values binned at 10 bp intervals and smoothed with a 3 bin moving average. Wildtype (WT) and *Δfun30* chromatin was digested with MNase and nuclease-protected DNA species sequenced using paired-end mode Illumina technology. Nucleosome sequencing data (nuc) traces were plotted as mirror images in the lower panel. The graph shows a map of the centre point positions of paired sequence reads with end-to-end distances of 150 bp+/−20% wild-type and *Δfun30* mutant chromatin samples surrounding *CEN1*. The frequency distributions, which effectively map chromatin particle dyads, were binned at 10 bp intervals, and smoothed by applying a 3 bin moving average. Peaks in the dyad distributions correspond to translationally-positioned nucleosomes in the original genome. The *CEN* core particle is also mapped using this method and can be visualised as a small peak centred on the *CEN* region marked with a grey box. Pink bars show the positions of ORFs (B–D) Genome browser plots of Fun30 ChIP-seq and nucleosome sequence distributions as described above for *CEN10, 11* and *12* respectively. Fun30-dependent changes in the height of a nucleosome dyad or *CEN* core particle peak are marked with a red asterix. Fun30-dependent changes in the position of a *CEN*-flanking nucleosome dyad peak are marked with red arrows. Genome browser plots for all yeast *CENs* are shown in Figure S6.

Because Fun30 has previously been shown to influence nucleosome positions at the *HMR* and *HML* boundaries [8], [9], we determined if Fun30 affects nucleosome positions at the centromere. In order to examine the role of Fun30 at all *CENs*, we used Illumina paired-end-mode technology to sequence micrococcal nuclease (MNase)-digested chromatin samples from wildtype and *Δfun30* mutant cells. Nucleosome-like particle positions were mapped as distributions of the center points of paired-end reads with an end-to-end distance of ∼150 bp. This class of size-selected paired-end reads largely defines the DNA entry- and exit-points on nucleosomes exposed by MNase digestion in the original chromatin sample. The frequency distributions of paired-read centre points, therefore, effectively estimate the frequency of nucleosome dyads, and peaks in the distributions correspond to translationally positioned nucleosome-like chromatin particles in the original genome [54], [55]. Figure 8 shows these data for areas around *CEN1*, *CEN10*, *CEN11*, and *CEN12*. At these sites both *CEN* flanking nucleosome positions, and/or the MNase accessibility of the *CEN* core particles themself are altered in the *Δfun30* mutant confirming that Fun30 is required for normal *CEN* chromatin structure. Figure S6 shows that such changes are seen at a majority of centromeres. Our analysis indicates a broad distribution of Fun30 over centromeres with peaks of several 100 bps or more, encompassing the central centromeric nucleosome (Figure 1, Figure S6), consistent with a role of Fun30 in regulating pericentromeric and centromeric chromatin. A similar localised alteration in nucleosome positioning in *Δfun30* cells was also observed at the other sites identified by Fun30 ChIP sequencing (Figure S7 shows Fun30-dependent nucleosome positioning at *ARS* elements). These results therefore suggest that Fun30 plays a major role in defining local nucleosome positioning at a variety of structural loci, particularly those with boundary and silencing functions, in a manner similar to its *S. pombe* ortholog Fft3 [11]. In conclusion, we show that Fun30 not only binds at centromeres but also affects their structure.

### *FUN30* Deletion Perturbs Htz1 Binding Genome-Wide, including at Centromeres

**Figure 9:**
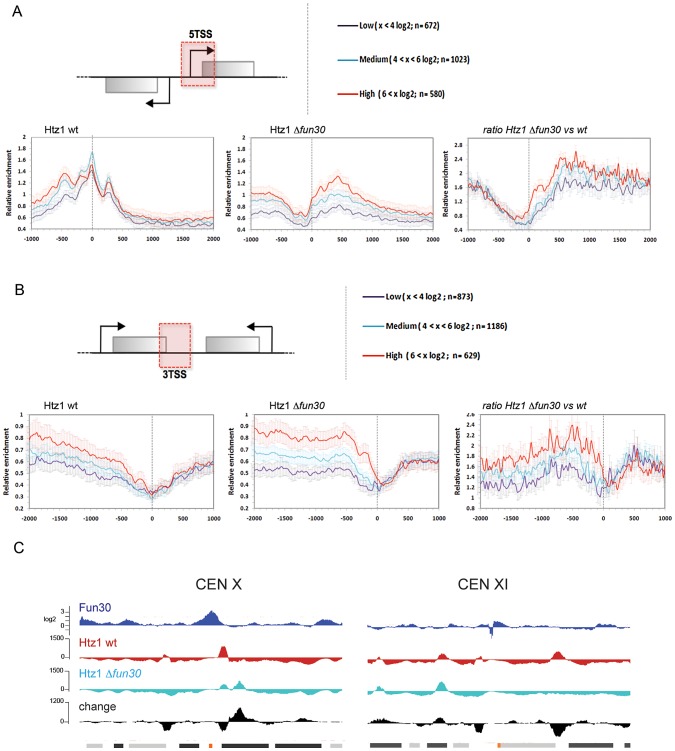
Fun30 affects Htz1 occupancy, including at centromeres. (A) Average occupancy analysis for histone Htz1 for divergent orientation genes relative to the 5′ Transcription start site (5TSS) position for wildtype cells (left panel), *Δfun30* cells (middle panel) and ratio *Δfun30* versus WT (right panel, *W303 3Myc-Htz1 versus W303 3Myc-Htz1 Δfun30*). (B) Average occupancy analysis for histone Htz1 for convergent orientation genes relative to the 3′ Transcription stop site (3TSS) position for wildtype cells (left panel), *Δfun30* cells (middle panel) and ratio *Δfun30* versus wildtype. (C) Effect of Fun30 on Htz1 occupancy 5 kbp up- and downstream of *CEN10* and *CEN11*. Shown is the Fun30 occupancy as measured by ChIP-seq in the top lane (dark blue, log_2_ scale, expressed as ratio of normalized sequence tag counts from ChIP to input). Htz1 occupancy from wildtype (wt, red) and *Δfun30* (light blue) are shown in the two lanes below expressed as normalized sequence tag counts corrected for input in linear scale. The change in occupancy of Htz1 is indicated in the lane below as the values from the *Δfun30* cells minus the values from wt cells (black). Positions of ORFs and centromeres are indicated in the lowest lane, orange box: centromere, back and grey boxes: ORFs in the sense and antisense direction, respectively.

Positioned nucleosomes at yeast promoters and other genomic sites, including areas in the vicinity of centromeres, telomeric elements and ARS, are often specifically enriched for Htz1 [56]–[60]. Fun30 has been shown to be able to catalyze histone H2A/H2B dimer exchange from nucleosomes in vitro including Htz1/H2B dimers [10]. Preliminary experiments suggested that *FUN30* deletion led to an increase of Htz1 at telomeric sites and within and around the silent mating locus *HMR* (ATV, WRW, PVW, data not shown). Therefore, we tested if *FUN30* deletion affected Htz1 occupancy genome-wide, including at centromeres. An analysis of Htz1 occupancy at divergent promoters allows focusing on the effect on promoters as opposed on terminator sites from adjacent genes. This shows a dramatic loss of Htz1 around that 5′ transcription start sites upon *FUN30* deletion and a corresponding increase within gene bodies (Figure 9A). A corresponding analysis of Htz1 occupancy surrounding terminator sites of convergent genes also demonstrates a drastic increase of Htz1 within the coding regions up to the 3′ terminator sites (Figure 9B). *FUN30* deletion does not affect the expression from any of the histone genes that we tested, including *HTZ1* (Figure S8) and did not affect total Htz1 protein levels relative to histone H3 (data not shown). We found changes in Htz1 occupancy around several centromeres, as shown for *CEN10* and *CEN11* in Figure 9C. While such changes are not seen at all centromeres they are evident at a majority of them (Figure S9). We see both loss of Htz1 occupancy at promoters in the vicinity of the core centromere and increased Htz1 binding at other, *e.g.*, downstream sites. This is well illustrated with *CEN10*, but also evident with other centromeres, such as in the vicinity of *CEN2*, *CEN4*, *CEN7*, *CEN11*, *CEN15*, and *CEN16*. Thus, Fun30 affects not only nucleosome positioning but also Htz1 occupancy at centromeres and this may be linked to defects in centromeric silencing that we observed upon *FUN30* deletion.

## Discussion

Fun30 is one of the most highly conserved members of the SWI/SNF-like enzymes and homologues appear to be present in all eukaryotes [4], [8]. However, its biological role and mode of function remained poorly characterized. In this study we employed genome-wide chromatin analysis to obtain insights into how Fun30 shapes the chromatin landscape. We show that loss of *FUN30* leads to alterations in nucleosome positions and occupancy at several sites that are normally occupied by Fun30, including centromeric and pericentromeric sites. Furthermore, deletion of *FUN30* leads to a substantial perturbation of the binding of Htz1, a key H2A-variant histone, and this is also observed around centromeres. We provide evidence that Fun30 is involved in supporting faithful chromosome segregation through its role in determining centromeric and pericentromeric chromatin. This role of Fun30 is required when centromeric function is perturbed, *e.g.*, by mutation of Cse4 or forcing transcription through centromeres. A recent study on a fission yeast homolog of Fun30, FFT3, shows a role for this protein in chromosome segregation and the regulation of CENP-A occupancy by promoting the formation of centromeric heterochromatin [11]. Unlike in fission yeast, budding yeast centromeres are not embedded in heterochromatin, but are surrounded by genes that are actively transcribed at some of the centromeres. Thus Fun30 has a role at centromeres that can be separated from a role in heterochromatin.

We found that Fun30 is required for normal nucleosome positioning and occupancy surrounding the centromeric nucleosome. There is also loss of nuclease protection over the centromeric nucleosomes at *CEN5*, *CEN9* and *CEN10* upon *FUN30* deletion. This loss may indicate a structural alteration of the centric nucleosome, maybe because of loss of a centromeric component, or a change in the overall chromatin configuration at this site that renders chromatin more accessible. In addition, and possibly linked to its role in determining nucleosome positioning, Fun30 is required for the correct occupancy of Htz1 genome-wide, including at centromeres. It is possible that the correct chromatin structure around the core centromeric nucleosome, including fine-tuned nucleosome spacing and correct Htz1 occupancy, is required for the optimal presentation of the centromere to the kinetochore. Thus, Fun30 may support centromere function by ensuring a correct chromatin environment. Because we detect an increase of transcription through *CEN3* upon Fun30 deletion, we believe that Fun30 may be involved in establishing a chromatin environment around the centromere that represses transcription over it, possibly by buffering against transcription emanating from surrounding genes. Both negative and positive roles for transcription have been reported at yeast centromeres [47], [49], [61]. The role of Fun30 in mediating correct Htz1 occupancy may therefore be linked to its role in suppressing transcriptional noise or in fine-tuning the precise level of transcriptional activity.

Fun30 appears to have a profound role in regulating Htz1 occupancy and this may be connected to its reported role in mediating silencing [8], [9]. Presently, we do not know if this is the result of direct chromatin remodeling by Fun30 or by a more indirect mechanism. For example, Fun30 may interact with and regulate components of the SWR1 complex that deposits Htz1 [62]–[64]. A direct role of Fun30 in regulating Htz1 occupancy would be consistent with its previously demonstrated *in vitro* histone dimer exchange activity, including H2AZ-H2B dimers [10]. It is intriguing that deletion of *FUN30* has a very similar outcome with respect to Htz1 occupancy as the deletion of chromatin remodeling factor complex *INO80*, which also results in a loss of Htz1 over promoters and gain of Htz1 occupancy downstream in the body of genes [65]. While there is evidence that Ino80 can regulate removal of Htz1 from nucleosomes directly [65], miss-incorporation of Htz1 on deletion of *FUN30* or *INO80* might be a common outcome of stress on the yeast cells. What could be the connection between Htz1 occupancy and centromere function? In fission yeast H2AZ mediates suppression of antisense transcripts [66]. It is possible that in budding yeast Htz1 also controls antisense transcripts, such as cryptic un-translated transcripts emanating from promoters and that this functions limits transcription into and over centromeres. Remarkably, H2A.Z has a role in mitosis in mammalian cells and is a structural component of mammalian centromeres [67], [68].

The recent study of fission yeast Fun30 homologue FFT3 showed a role of this factor at boundary elements by evicting nucleosomes and preventing the spread of euchromatin into heterochromatin. We also found that Fun30 accumulates at putative boundary elements, such as tRNA genes (this study, [8]). Therefore, it is likely that budding yeast Fun30 has a similar role as proposed for FFT3, and this may, at least in part, be linked to the silencing defects in *fun30*-deleted cells that we observed previously [8].

While we did not find that deletion of *FUN30* affected binding of Cse4 over the endogenous *CEN3* and an inducible *CEN*, we show that Fun30 affects centromeric and pericentromeric chromatin (Figure 8, Figure 9) in line with its role in supporting chromosome segregation (Figure 3, Figure 4, Figure 5). Even the relatively simple centromere of budding yeast is a very complex, multi-subunit structure that, on top of this, is highly dynamic. While Cse4 is an essential component of the centromere, centromere function can be compromised at several levels, including the pericentromeric chromatin. The studies of the diverse roles of ATP-dependent nucleosome remodeling factors in supporting centromere function, as described below, make this point very clearly. Several other ATP-dependent nucleosome remodeling factors have been implicated in chromosome segregation and centromere maintenance or function in budding yeast, including the RSC complex [69]–[71] and the SWI/SNF complex [26]. RSC has been proposed to act following Cse4 recruitment and SWI/SNF has been shown to support segregation by preventing Cse4 binding to extra-centromeric sites [26], [70]. The budding yeast Ino80 complex also binds centromeres and is involved in sister chromatid cohesion, but is not required for centromeric association of kinetochore components including Cse4 [72]. In fission yeast, HRP1, a homolog of the budding yeast Chd1 protein, is required for faithful chromosome segregation and full CENP-A (CNP1) occupancy [73], [74]. Similar conclusions have been made for chicken and human Chd1 [75], but it has also been reported that Chd1 has no role in CENP-A binding in *Drosophila* [76]. It is not known if the budding yeast Chd1 fulfills a centromere function. Overall, a picture emerges where several remodeling factors, including Fun30, have complementary and overlapping roles in assuring correct centromere and pericentromeric chromatin structure and faithful chromosome segregation. Whether a remodeling factor exists in budding yeast that is actively involved in depositing Cse4 is an open question. Recent work from the Bloom laboratory highlights the importance of regulated histone dynamics of the pericentromeric chromatin for chromosome segregation by maintaining kinetochore structure during mitosis and implicates remodeling factors in this process [77].

ISW2 is a nucleosome remodeling factor that prevents noncoding transcription away from promoters and other nucleosome depleted regions, by limiting nucleosome free region size [78]. Fun30 may be another remodeling factor that regulates noncoding transcription. We detected an increase of nongenic transcripts by qPCR over centromeres on deletion of *FUN30*. However, using northern blotting upon *FUN30* deletion we did not detect an increase of cryptic unstable transcripts (CUTs) at several other sites including at sites between convergent genes where we find peaks of Fun30 binding (JRM, unpublished results). Given the pronounced Fun30 binding over intergenic regions, especially between convergent genes and its link to loss of histone H3, it will be interesting to examine what is the biological role of Fun30 at these sites. A clue may be given by the fact that Fun30 also binds intergenic sites, tRNA elements, ARS sequences, snoRNA genes and centromeres. All these sites have been shown to also bind cohesin and condensin [79], [80]. Future studies will examine if Fun30 collaborates with these chromosome-organizing factors and elucidate how Fun30 identifies its specific binding sites, such as centromeres.

## Materials and Methods

### Cell Cultures, Plasmids

Yeast strains used in this study are listed in Table 1. Strains SC138 and SC140 were generated by integrating CSE4-myc13 driven by the *CSE4* promoter into the *LEU2* locus of strains KBY4001B and SC110 using BstX1 cleaved integration vector SB500, kind gift from Dr Sue Biggins. Standard budding yeast genetic techniques and media were used according to Guthrie *et al.* [81]. Cells were usually grown in YPD media at 30°C. For spotting and serial dilution experiments, cells were grown to mid-log phase and counted by haemocytometer. Cultures were diluted to 2.5×10^6^ cells/ml with sterile H_2_O, than 1∶5 serial dilutions were performed five times. For the dicentric chromosome assay, strains containing *GALCEN3* were plated for single colonies on YP galactose or glucose at 30°C as described in [46]. For the mitotic stability assay, cells transformed with pUG25 centromere plasmid (Gueldener and Hegemann, unpublished, [82]) were grown in nonselective minimal media for 12 generations and then plated on −leu or + leu plates. Plasmids used in this study are listed in Table 2.

**Table 1:** *S. cerevisiae* strains used in this study.

Strains	Mating type	Genotype	Source/Reference
**Y00000 (BY4741 )**	MAT a	*his3Δl leu2Δ10 met15Δ ura3Δ0*	EUROSCARF
**Y00389**	MAT a	*his3Δl leu2Δ10 met15Δ ura3Δ0 YAL019W::kanMx4*	EUROSCARF
**SC13**	MAT a	*his3Δ1; leu2Δ0; met15Δ0; ura4Δ0; trf4Δ::natMX6*	Jon Houseley
**SC15**	MAT a	*his3Δ1; leu2Δ0; met15Δ0; ura4Δ0; trf4Δ::natMX6 YAL019W::kanMx4*	This study
**SBY617**	MAT a	*ura3-1 leu2,3-112 his3-1 trp1-1 ade2-1 can1-100 Δbar1 CSE4-12myc::URA3*	[23]
**AHY666**	MAT α	*ade2-101 his3-del200 lys2-801 trp1-Δ63 ura3-53 leu2-3, 112 cse4-1 (lys2+?)*	[95]
**SC39**	MAT a	*his3Δ1; leu2Δ0; met15Δ0; ura4Δ0 CSE4-12myc::URA3 YAL019W::kanMx4*	This study
**SC53**	MAT α	*ade2-101 his3-del200 lys2-801 trp1-del63 ura3-53 leu2-3, 112 cse4-1 (lys2+?),YAL019W::kanMx4*	This study
**SC56**	MAT a	*ura3-52, trp1Δ2, leu2-3_112, his3-11, ade2-1 can1-100 pYES2.1*	This study
**SC58**	MAT a	*ura3-52, trp1Δ2, leu2-3_112, his3-11, ade2-1 can1-100 YAL019W::kanMx4 pYES2.1*	This study
**SC60**	MAT a	*ura3-52, trp1Δ2, leu2-3_112, his3-11, ade2-1 can1-100 YAL019W::kanMx4 pFA1*	This study
**SC62**	MAT a	*ura3-52, trp1Δ2, leu2-3_112, his3-11, ade2-1 can1-100 YAL019W::kanMx4 pFA3*	This study
**SC64**	MAT a	*ura3-52, trp1Δ2, leu2-3_112, his3-11, ade2-1 can1-100 YAL019W::kanMx4 pFA5*	This study
**SC66**	MAT α	*ade2-101 his3-del200 lys2-801 trp1-del63 ura3-53 leu2-3, 112 cse4-1 (lys2+?) pYES2.1*	This study
**SC68**	MAT α	*ade2-101 his3-del200 lys2-801 trp1-del63 ura3-53 leu2-3, 112 cse4-1 (lys2+?),YAL019W::kanMx4 pYES2.1*	This study
**SC70**	MAT α	*ade2-101 his3-del200 lys2-801 trp1-del63 ura3-53 leu2-3, 112 cse4-1 (lys2+?),YAL019W::kanMx4 pFA1*	This study
**SC72**	MAT α	*ade2-101 his3-del200 lys2-801 trp1-del63 ura3-53 leu2-3, 112 cse4-1 (lys2+?),YAL019W::kanMx4 pFA3*	This study
**SC74**	MAT α	*ade2-101 his3-del200 lys2-801 trp1-del63 ura3-53 leu2-3, 112 cse4-1 (lys2+?),YAL019W::kanMx4 pFA5*	This study
**KBY4001B**	MAT a	*ade1 met14 ura3-52 leu2 his3 his4::GALCEN3URA3*	[46]
**KBY4005**	MAT a	*ade1 met14 leu2 his3 his4::GALCEN3URA3 chl4::KANr*	[46]
**SC110**	MAT a	*ade1 met14 leu2 his3 his4::GALCEN3URA3 YAL019W ::NatMx6*	This study
**SC138**	MAT a	*ade1 met14 leu2 his3 his4::GALCEN3URA3 LEU2::CSE4prom-CSE4-12myc*	This study
**SC140**	MAT a	*ade1 met14 leu2 his3 his4::GALCEN3URA3 YAL019W ::NatMx6 LEU2::CSE4prom-CSE4-12myc*	This study
**SC98**	MAT a	*his3Δl leu2Δ10 met15Δ ura3D0 pUG25 (Leu2+)*	This study
**SC99**	MAT a	*his3Δl leu2Δ10 met15Δ ura3D0 pUG25 (Leu2+)*	This study
**SC100**	MAT a	*his3Δl leu2Δ10 met15Δ ura3D0 pUG25 (Leu2+)*	This study
**SC101**	MAT a	*his3Δl leu2Δ10 met15Δ ura3D0 YAL019W::kanMx4 pUG25 (Leu2+)*	This study
**SC102**	MAT a	*his3Δl leu2Δ10 met15Δ ura3Δ0 YAL019W::kanMx4 pUG25 (Leu2+)*	This study
**SC103**	MAT a	*his3Δl leu2Δ10 met15Δ ura3Δ0 YAL019W::kanMx4 pUG25 (Leu2+)*	This study
**SLY806**	MAT alpha	*MATα PHIS3-GFP-LacI2-HIS3, LacO256:LEU2, URA3-CHRIII116000, TRP1:GALpr at CEN3, ade2-1, leu2-3,112, trp1-1, can1-100*	[45]
**SC117**	MAT alpha	*MATα PHIS3-GFP-LacI2-HIS3, LacO256:LEU2, URA3-CHRIII116000, TRP1:GALpr at CEN3, ade2-1, leu2-3,112, trp1-1, can1-100 Δfun30:kanMx6*	This study
**W303 3Myc-Htz1**			[57]
**W303 3Myc-Htz1 ** ***Δfun30***			This study

**Table 2:** Plasmids used in this study.

Name	Marker/Backbone	Source/Reference
**pYES2.1**	pYES2.1 V5- TOPO (ura3+)	Invitrogen
**pFA1**	pYES2.1/*FUN30 (ura3+)*	[8]
**pFA3**	pYES2.1/*FUN30-*ATPase(ura3+)	[8]
**pUG25**	CEN6/ARSH4(Leu2+)	Gueldener and Hegemann, unpublished
**SB500**	CSE4-myc13 integrating plasmid markedWith LEU2 based on pSB236	Sue Biggins

### RNA Extraction and Reverse Transcription (RT)

For RT-qPCR analysis, total RNA was extracted from mid-logarithmic phase cells (O.D._600_: 0.7) in YPD media using the hot acidic phenol standard extraction protocol [83]. Total RNA was treated by DNAse I amplification grade (Invitrogen). For analysis by RT-qPCR, RNA was reverse transcribed and amplified in one-step using specific primers and iScriptTM One-Step RT-PCR Kit with SYBR Green (Bio-Rad Laboratories). Each sample was prepared in duplicate and a control without the Reverse Transcriptase was included to control for contaminating DNA.

### Chromosome Segregation Assay and Microscopy

Cells were grown overnight in minimal media without uracil and leucine containing 2% glucose. Cells were then collected, washed three times in minimal media without glucose and grown in minimal media with either 2% glucose or 3% galactose for 4 h. To count the number and location of GFP dots of the LacO array proximal to *CEN3* [45], cells were fixed at room temperature with 2% paraformaldehyde, 10 min directly in the media and then washed once with PBS. A Nikon Eclipse E600 equipped with a ×100 1.4 NA lens (Nikon), GFP filter, a Cascade 512B digital camera (Photometrics) and MetaMorph software (Universal Imaging Corporation) was used to determine the number of GFP dots per cell by moving the focal plane through the sample and analyzing the live digital image on the computer screen.

### Chromatin Immunoprecipitation

ChIP was carried out essentially as described [8]. Overnight cultures grown in YPD at 30°C were diluted to 0.2 OD_595_, then grown to 0.7 OD_595_ at 30°C before crosslinking. Samples were crosslinked 15 min for H3, 3Myc-Htz1 and Cse4-Myc or 30 min for Fun30 detection with 1% final formaldehyde and chromatin extracts were sonicated to ∼500 bp. Triplicates or duplicate ChIP samples were validated by qPCR. Chromatin extracts were then immunoprecitated with 5 µg the Rabbit polyclonal anti-H3 (Ab1791, Abcam) for histone H3; 2 µg affinity-purified rabbit polyclonal anti-Fun30 for Fun30 [8] or with 2 µg of mouse monoclonal anti-myc (9E10, Ab32, Abcam) for Cse4-Myc and 3Myc-Htz1.

### Quantitative PCR (qPCR) Analysis

Immunoprecipitated and Input DNAs were analysed by qPCR using the SYBR Green PCR Master Mix (Applied Biosystems). For immunoprecipitated DNA a 8-fold dilution was performed, input DNA was diluted 500 times; primers used are listed in Table 3. The enrichment of the protein in a specific locus was calculated as percentage of input DNA. The background binding was calculated form the no-antibody control and subtracted from the respective sample.

**Table 3:** Oligonucleotide primers used in this study.

Primer Name	Sequence 5′-3′
**FUN30 Upstream**	TACAAGCCTGTTATAGCCTTTAATGATCAC
**FUN30 Downstream**	CCATTTCTCTCCCCAGATTAAA
**FUN30TAP fwd**	GACAAGCTGCTGATAGGGCAC
**FUN30TAP rev**	GTTCACCATTTCTCTCCCCAG
**KANMX downstream**	TGATTTTGATGACGAGCGTAAT
**CEN I Forward Camahort et al. 2009, also RT-qPCR**	TGACATTGAACTTCAAAACCTTT
**CEN I Reverse Camahort et al. 2009, also RT-qPCR**	GGCGCTTGAAATGAAAGCTC
**PM70 (CENIII) Camahort et al. 2009**	AGTGTCTTCGCATAAAATCCAG
**PM71 (CENIII) Camahort et al. 2009**	CATCTATTTACTGCTATTAAGCG
**PM72 (CENIII) Camahort et al. 2009**	CATACCATGCTTTGTTATCGTC
**PM73 (CENIII) Camahort et al. 2009**	ATTTTATGCGAAGACACTGCTG
**PM74 (CENIII) Camahort et al. 2009**	CATCTTTGAAAAGTTCATCAAGG
**PM75 (CENIII) Camahort et al. 2009**	CGATAACAAAGCATGGTATGGC
**PM76 (CENIII) Camahort et al. 2009**	ATATTGTTTGGCGCTGATCGC
**PM77 (CENIII) Camahort et al. 2009**	CTTGATGAACTTTTCAAAGATGAC
**PM22 (CENIII) Camahort et al. 2009, also RT-qPCR**	GATCAGCGCCAAACAATATGG
**PM48 (CENIII) Camahort et al. 2009, also RT-qPCR**	AACTTCCACCAGTAAACGTTTC
**PM78 (CENIII) Camahort et al. 2009**	GTCAACGAGTCCTCTCTGGC
**PM79 (CENIII) Camahort et al. 2009**	TTTACTGGTGGAAGTTTTGCTC
**PM80 (CENIII) Camahort et al. 2009**	GAATATGATAATGGTTACACCAG
**PM81 (CENIII) Camahort et al. 2009**	GAGAGGACTCGTTGACGTAG
**PM82 (CENIII) Camahort et al. 2009**	GATTTAATGCACGTTATGTTTCG
**PM83 (CENIII) Camahort et al. 2009**	TGTAACCATTATCATATTCATGAC
**PM84 (CENIII) Camahort et al. 2009**	GTAAGAGGTAGGTTTTGCAGG
**PM85 (CENIII) Camahort et al. 2009**	ATAACGTGCATTAAATCTCACTG
**GAL2orfF Camahort et al. 2009**	CGAACTCAGTTCAATGGAGAGT
**GAL2orfR Camahort et al. 2009**	TACCGGCCATGATCAGATCT
**ARS315_F**	GCGCGTCAACTTTCTACCA
**ARS315_R**	ATTTTCTTGGCGCTACGATG
**snR35_F**	GTCCTACCAGCCCTTGCATA
**snR35_R**	CAAGTCCATCGGAGAGATCA
**Control1_F**	TCGCAAAGAGATAATGGTGCT
**Control1_R**	TTTCGATGTCGTCAGCAGTC
**Control2_F (also used for RT-qPCR)**	TAGCACGGTGCATCAGAAAG
**Control2_R (also used for RT-qPCR)**	CGCTACCAATACCAGGGAAA
**Control3_F**	GATGAGGCAACCAAGAAGGA
**Control3_R**	TCGTAGCGTGGCATAAAACA

### ChIP–Seq Library Preparation

Illumina sequencing was performed using protocols derived from [84]–[86] and the standard Illumina protocol according to the manufacturer. ChIP DNA fragments were purified and concentrated using MinElute columns (QIAGEN). Eluted DNAs from two pooled ChIP reactions of biological replicas (equal amount of DNA) were separated by electrophoresis through 2% agarose in TAE and DNA fragments with size range 150–450 bp were excised. Excised DNA fragment were purified using the QIAGEN Gel Extraction Kit and eluted in 30 µl of EB buffer (10 mM Tris-HCL, pH 8). The entire size selected ChIP reaction was then used in the end-filling and A-tailing reactions, essentially as described in the standard Illumina protocol, using standard molecular biology reagents purchased from New England Biolabs. The adapter ligation step was performed using barcoded single-end adapters synthesized by Sigma-Genosys described in [86]. Briefly, forward and reverse adapters were mixed in equimolar ratios, incubated at 95°C for 5 minutes, and allowed to anneal by using a ramp of −1°C/10 seconds until the sample reached 4°C. The ligation of adapters with DNA fragments was performed using T4 DNA ligase from Enzymatics, with incubation for 30 minute at 16°C followed by an additional 30 minutes at 22°C. Next the library was purified using the QIAGEN MinElute kit and separated in a 2% agarose/TAE gel for 1 hour at 90 V. Libraries were excised from the gel between 150 bp and 500 bp. Amplification was performed using *Pfx* Platinum polymerase (Invitrogen) for 15 cycles as described by Quail et al. [84]. The libraries were concentrated with QIAGEN MinElute kits and electrophoresed in a 1.9% agarose/TAE gel. Samples were quantified with SYBR Green qPCR Master Mix (Applied Biosystems) and the primers SYBR FP4 and SYBR RP7 and compared to a standard curve of phiX174 library, as described [84]. The libraries were diluted to 10 nM in EB buffer.

### RNA–Seq Library Preparation

Overnight cultures were grown in YPD at 30°C were diluted to 0.2 OD_595_, then grown to 0.7 OD_595_ at 30°C. Total RNA was isolated using the hot acidic phenol method [83]. Next 10 µg aliquots were treated with DNase I amplification grade (Invitrogen) for 30 minutes at 37°C, purified by ethanol precipitation and quality-checked by 8 M Urea 6% polyacrylamide gel electrophoresis in 0.5× TBE. PolyA RNA were purified using Oligo-dT Dynabeads (Invitrogen). Purified polyA RNA samples were concentrated by ethanol precipitation and then fragmented using the Ambion RNA fragmentation kit. Samples were ethanol precipitated and the RNA was used in first strand and second strand cDNA synthesis with random hexamers at 150 ng/µl. The entire reaction was used in library generation. Libraries are summarized in Table 4.

**Table 4:** Summary of sequence libraries.

libraries	Total Read Count	Fold Coverage	References
Input	35 047 745	100	This study
ChIP FUN30	4 881 253	14	This study
ChIP H3 WT	8 610 178	24	This study
ChIP H3 *Δfun30*	8 610 631	24	This study
WT cDNA	4 506 311	12	This study
*Dfun30* cDNA	4 886 511	13	This study
ChIP Htz1 WT	1 937 248	5	This study
ChIP Htz1 *Δfun30*	2 402 571	6	This study
Nuc.-seq WT	13 626 902 read pairs	195 reads/nucleosome	This study
Nuc.-seq *Δfun30*	13 362 948 read pairs	195 reads/nucleosome	This study
Input (for Cse4)	10 523 511	25	[86]
ChIP Cse4	2 184 703	5	[86]

### ChIP–Seq Data Analysis

To increase the sequence yields, the Illumina sequence reads, carrying custom barcodes at the start, were re-analyzed using bareback-processing [87]. Barcodes were used to sort files and were subsequently stripped off. Alignments to the yeast genome (genome build SGD1.01, Dec 2006) were performed with Bowtie [88] using default options and ‘–best’. Next, data were loaded into Seqmonk (http://www.bioinformatics.bbsrc.ac.uk/projects/seqmonk/). Read quantification of probe regions were designed depending on purpose. For analysis of 5′ Transcription Start Site (5TSS) and 3′ Transcription Termination Site (3′TTS) regions, UTRs length were obtained from [89]. Tiled probes of 25 bp resolution were generated from −1000 to +2000 bp relative to the 5′TSS region of the gene and from −2000 to +1000 bp relative the 3′TTS regions. Quantification of the reads were corrected for the total read count and for probe length. Normalizations were performed using an input DNA library. For the analysis of 5′ intergenic regions (5′IGRs), genomic elements and 3′ intergenic regions (3′IGR) we performed gene annotations according to Figure S10. For coding genes having identified 5′UTRs and 3′UTRs according to [89], 5′IGR and 3′IGR regions comprised UTR regions plus an extended regions of 150 bases to integrate the promoter or terminator regions. Each segment was then averaged vertically for each subgroup of expression to create the average binding values along each position. Three expression categories were assigned according to their log_2_ signal intensities. Visualization of data was performed using the Affymetrix Integrated Genome Browser (IGB) (http://www.affymetrix.com/) and Mochiview [90]. The resulting ratio ChIP versus DNA input (from chromatin) were extracted for each probe position, defined as the center (6^th^) base coordinate for each 13-nucleotide probe. High-resolution cluster visualization of 5′TSS and 3′TTS were performed using MultiExperiment Viewer MeV4.5.1 [91], [92]. Correlations were performed using Venn Mapper software (http://www.gatcplatform.nl/vennmapper/) [93]. GO analysis was performed using Mochiview with multiple testing correction [90]. Annotation features were downloaded from SGD database (http://www.yeastgenome.org/), genome version SGD01.01 (Dec 2006). For determining gene orientation we also considered noncoding genomic elements, *i.e.* snoRNAs, snRNAs and tRNAs

### Nucleosome Sequencing

*S. cerevisiae* used for nucleosome sequencing were EUROSCARF collection wild-type reference strain BY4742 (*MATα*; *his3Δ1*; *leu2Δ0*; *lys2Δ0*; *ura3Δ0*) and mutant Y10389 (*MATα*; *leu2Δ0*; *lys2Δ0*; *ura3Δ0*; *Δfun30::KanMX4*). Cells were grown in YPD rich medium (1% peptone, 1% yeast extract, 2% D-glucose) at 29°C to 2.6–2.8×10^7^ nucleated cells per ml (determined by haemocytometry). Chromatin digestion and DNA preparation was performed exactly as described [55]. Briefly, un-fixed detergent-permeabilised yeast spheroplasts were incubated with MNase, and then a DNA fraction containing all MNase-digested DNA species <1 Kb (including sequences protected by sequence-specific DNA binding proteins, mono-nucleosomes and poly-nucleosomes) was released and purified. 10 µg pooled triplicate samples of DNA (Figure S11A) were processed for library preparation, size-selected on polyacrylamide gels (to preserve the size distribution of DNA fragments) and sequenced using the 76 base Illumina GAIIx paired end mode process exactly as described [55]. Raw paired sequence reads are deposited at the NCBI short read archive under accession number SRA039099.2. Paired reads were aligned to the NCBI *S. cerevisiae* reference genome using Bowtie 0.12.7 [88] with command line flags: -n 0 –trim3 40 –solexa.3-quals –maxins 5000 –fr -k 1 –sam. Sequences were, therefore, clipped from the 3′ end to 36 bp allowing Bowtie to return overlapping read pairs resulting from sequencing of relatively short input DNA species. 13,626,902 and 13,362,948 perfectly-aligned reads pairs were obtained from the wild-type and *Δfun30* samples respectively. The paired reads were sorted into a range of classes based on the SAM format ISIZE value (difference between 5′ end of the mate read and the 5′ end of the first mapped read) plus or minus a window value of 0.2 times ISIZE as described [55]. Figure S11B shows the frequency distributions of aligned paired sequence reads from both yeast strains, and confirms that the ISIZE distributions reflect the ∼150 bp nucleosomal periodicity of the input chromatin samples. To specifically map nucleosomes, aligned paired sequence reads with an ISIZE of 150 bp±30 bp were selected, the assumption being that the DNA species falling into this size class would have been generated by protection of DNA from MNase digestion in chromatin by mono-nucleosome binding. The center value of each read-pair was calculated to represent the map position of the putative nucleosome dyad, and a genome-wide frequency distribution of the dyad positions determined and binned to 15 bp. The frequency distributions were smoothed by taking a 3 bin moving average and output in a zero-referenced, chromosome base, three-column format (chromosome number, genomic bin position, dyad frequency value) as described [55]. The frequency-distribution files for wild-type and *Δfun30* mutant cells were given an .sgr file ending and rendered using the Integrated Genome Browser [94] to produce the nucleosome dyad frequency traces presented in this work.

### Accession Code

ChIP-seq and RNA-seq read data has been deposited in the ArrayExpress database (http://www.ebi.ac.uk/arrayexpress/) under accession codes E-MTAB-955, E-MTAB-956 and E-MTAB-759. Raw paired sequence reads of nucleosome mapping are deposited at the NCBI short read archive, accession number SRA039099.2.

## Supporting Information

Figure S1 Fun30 preferentially binds intergenic regions over coding regions (ORFs). A) Analysis of Fun30 binding to intergenic regions, red line: ratio of 5′ intergenic *versus* ORF regions, blue line: ratio 3′ intergenic *versus* ORF regions. B) Fun30 shows a pronounced binding at the intergenic 3′ end region and this enrichment is directly correlated with expression levels, shown is the average gene analysis for Fun30 binding in relation to RNA transcript levels (determined by RNA-seq in this study) number of genes (n) in each category is indicated in the figure, error bars: 95% confidence interval. C) left panel: Binding profile of Fun30 relative to 5 Transcriptional Start Sites (5′TSS). The clusters contain 4560 genes where the 5′TSS has been identified [89]. Grey bars on right side indicate respectively expression level for each gene and promoter orientation (TP: Tandem Promoters, CP: Convergent Promoters). Values are represented in log2; right panel: Binding profile of Fun30 relative to 3′ Transcriptional Termination Sites (3′TTS). The clusters contain 5208 genes with identified 3′TTS [89]. Grey bars on right side indicate respectively expression level for each gene and promoter orientation (CT: Convergent Terminators, TT: Tandem Terminators). Values are represented in log2. D) As in (C), right panel, but corresponding histone H3 occupancy in wildtype cells and the change in histone H3 occupancy as ratio of occupancy between *fun30*-deleted/wildtype cells is shown.
(TIF)

Figure S2 Fun30 is enriched over centromeric regions. A) Validation of Fun30 binding sites by ChIP following qPCR. Fun30 binding at control regions (Ctrl1–3) where Fun30 did not bind according to our ChIP-seq data, and binding to *CEN1*, snR35, ARS315. B) Fun30 binding to *CEN3* and surrounding region using primer pairs spanning +/−2 kb, controls as in (A). Level of enrichment is expressed as % DNA precipitated as compared to input. Background binding to beads was substracted, error bars represent the difference of the maximum and minimum values from the mean. Shown are results from 2 biological replicas, each with 2 technical replicas.
(TIF)

Figure S3 Fun30 primarily acts as a repressor of transcription. Moving average plot (window size = 150 genes, step size = 1 gene) of the mRNA transcription level ratios in Y00389 (*Δfun30*) versus BY4741/Y00000 (WT) plotted as a function of mRNA levels in WT at 30°C in YPD (normalized reads intensity by bases pairs count ×1000/divided by gene length, [27]). The gene expression ratio from *Δfun30 versus* wildtype was plotted as moving average of this ratio as a function of the wildtype gene expression levels. The global expression profile for the *Δfun30* mutant showed a ratio of 1 for moderately expressed and highly expressed genes indicating that Fun30 does not affect genes at this range of expression levels. Silent or weakly expressed genes in wildtype showed a high median expression ratio.
(TIF)

Figure S4 Deletion of Fun30 does not affect promoter activity of *GAL1* integrated at centromere *CEN3*. Analysis of *GAL1* promoter induction at *CEN3* locus. RNA from SLY806 (control, black) and SC117 (*Δfun30*, dashed gray) strains was isolated at indicated time points after addition of galactose and analyzed by RT-qPCR using primers specific for *CEN3* locus (PM22/PM48).
(TIF)

Figure S5 Fun30 regulates histone H3 occupancy at intergenic regions. A) 3′ regions of genes show the greatest enrichment for Fun30 when compared to promoter or coding regions. Average occupancy of Fun30 for divergent orientation promoters (left panel) and convergent terminators (right panel). The data were binned into three groups (High, medium, low) according to the expression level of wildtype cells. The number of genes (n) in each category is indicated. Error bars represent 95% confidence intervals. The genomic region of interest was divided into 40 equally sized bins. The 5′ and 3′ flanking regions have 1250 bp from respectively the beginning and the end site of the genomic elements and divided into a 50 fragments of 50 bases (see Materials and Methods for full description). (B, C) Average trends in specific promoter or terminator regions - *i.e.* only divergent or convergent genes - were determined for the histone H3 occupancy profiles in wildtype and *Δfun30* cells. As previously shown, histone H3 is mainly present within coding regions whereas in both promoter and terminator specific regions histone H3 is relatively depleted. In *Δfun30* mutants there is an increase of histone H3 at promoter and terminator regions. B) Average occupancy analysis for histone H3 for divergent orientation genes relative to the 5′TSS position for wildtype cells (left panel) and ratio *Δfun30* versus WT (right panel, Y00389 *versus* BY4741/Y00000). C) Average occupancy analysis for histone H3 for convergent orientation genes relative to the 3′TTS position of genes for wildtype cells (left panel) and ratio *Δfun30* versus WT (right panel, Y00389 *versus* BY4741/Y00000). D) Venn diagrams showing results of hypergeometric probability tests for 5′IGR and 3′IGR Fun30 targets and changes in histone H3 in *Δfun30* mutant. This analysis revealed that the changes in histone H3 occupancy are significant for the 5′ and 3′ intergenic regions. Venn diagram illustrating the overlaps in 5′ intergenic regions (5′IGR) and 3′ intergenic regions (3′IGR) between the genes that display increased Fun30 binding (1.5 fold up enrichment) and increased histone H3 occupancy in the *Δfun30* mutant. Hypergeometric probability values are indicated. For promoter (divergent genes) or terminator specific regions (convergent genes) these are respectively *P(X = 31)* = 1.77×10^−9^ and *P*(X = 156) = 5.43×10^−8^.
(TIF)

Figure S6 The majority of yeast *CENs* exhibits Fun30-dependent changes in flanking nucleosome position and/or *CEN* core MNase accessibility. A) Genome browser traces of Fun30 ChIP enrichment and nucleosome dyad frequency centered on and surrounding yeast *CEN1*; B) *CEN2*; C) *CEN3*; D) *CEN4*; E) *CEN5*; F) *CEN6*; G) *CEN7*; H) *CEN8*; I) *CEN9*; J) *CEN10*; K) *CEN11*; L) *CEN12*; M) *CEN13*; N) *CEN14*; O) *CEN15*; P) *CEN16*. The upper trace of each panel shows log_2_ Fun30 ChIP enrichment values binned at 10 bp intervals and smoothed with a 3 bin moving average. The wild-type (WT) and *Δfun30* mutant nucleosome (nuc) traces were plotted as mirror images in the lower panel. Centre point positions of paired sequence reads with end-to-end distances of 150 bp+/−20% were mapped across the yeast genome for the wild-type and *Δfun30* mutant MNase-digested chromatin sequencing samples, binned at 10 bp intervals, and the resulting frequency distributions smoothed by applying a 3 bin moving average. This class of size-selected paired-end sequence reads largely defines the DNA entry- and exit-points on nucleosomes exposed by MNase digestion in the original chromatin sample. The frequency distributions of paired-read center points therefore effectively estimates the frequency of nucleosome dyads [54], [55] and peaks in the distribution correspond to translationally positioned nucleosomes in the original genome. The *CEN* core particle is also mapped using this method and can be visualized as a small peak centered on the *CEN* region marked with a grey box on each browser panel, pink boxes mark the surrounding ORFs. Fun30-dependent changes in the height of a nucleosome dyad or *CEN* core particle peak are marked with a red asterix. Fun30-dependent changes in the position of a *CEN*-flanking nucleosome dyad peak are marked with red arrows.
(TIF)

Figure S7 Fun30 is required for normal nucleosome positioning at other sites identified by ChIP-seq. A. *ARS* regions show Fun30-dependent nucleosome positioning surrounding the *ARS* consensus sequence (ACS). The upper graph shows a plot of the cumulative Log_2_ Fun30 ChIP enrichment values centered on the yeast ACS elements defined by Nieduszynski *et al.* [96]. The lower graph shows plots of the cumulative nucleosome dyad frequencies in the same region for wildtype (black line) and *Δfun30* mutant (red line) chromatin sequencing data sets. The cumulative nucleosome dyad frequencies in each 15 bp bin were normalized by dividing by the average nucleosome dyad frequency for the whole feature window in order to place “random” nucleosome occupancy at a value of 1. Changes in cumulative distribution between wildtype and the *Δfun30* mutant are indicated with asterisks. B. Genome browser trace of nucleosome dyad frequencies at Fun30-dependent *ARS202* plotted as described for *CEN* analyses, Figure 8, Figure S6. Three nucleosomes surrounding the *ARS* which exhibit Fun30-dependent changes in position are marked with arrows.
(TIF)

Figure S8 RNA-seq analysis shows that histone gene expression is not changed upon *FUN30* deletion. Expression in wildtype cells: dark grey bars, in *Δfun30* cells: light grey.
(TIF)

Figure S9 Fun30 affects Htz1 occupancy around a majority of centromeres. Effect of Fun30 on Htz1 occupancy 5 kb up- and downstream of *CEN1–16*. Shown is Htz1 occupancy from wildtype (wt, red) and *Δfun30* cells (light blue, below) expressed as normalized sequence tag counts corrected for input in linear scale. The change in occupancy of Htz1 is indicated in the lane below as the values from the *Δfun30* cells minus the values from wt cells (black). Positions of ORFs and centromeres are indicated in the lowest lane, orange box: centromere, back and grey boxes: ORFs in the sense and antisense direction, respectively. Axis and scales as in Figure 6.
(TIF)

Figure S10 Description of flanking regions annotations for coding gene. A) 5′IGR and 3′IGR region assignment for genes having identified 5′UTR and/or 3′UTR by [89]. B) 5′IGR and 3′IGR region assignment for gene having unidentified 5′UTR and/or 3′UTR.
(TIF)

Figure S11 MNase digested chromatin samples processed for paired-end mode Illumina DNA sequencing. A. DNA from MNase digested chromatin fractions purified from wild-type and *Δfun30* yeast strains separated by agarose gel electrophoresis and stained with ethidium bromide. B. Graph of the number of aligned paired-end reads obtained by Illumina GAIIx sequencing of material shown in Fig. 1A versus paired-read end-to-end distance (SAM format ISIZE value). Peaks at ∼150 bp, 300 bp and 450 bp are marked and correspond to mono-, di- and tri-nucleosome DNA fractions respectively. The end-to-end distances of paired sequence reads therefore reflect the distribution of chromatin particle input DNA.
(TIF)

Table S1 This EXCEL spreadsheet based table provides gene lists of up- and down-regulated genes in *fun30-*deleted cells, a list of genes that genetically interact with *FUN*30.
(XLS)
