# Supplementary figures and images for: SWI/SNF-Like Chromatin Remodeling Factor Fun30 Supports Point Centromere Function in S. cerevisiae
Source: PLoS Genet. 2012 Sep 27;8(9):e1002974. doi: 10.1371/journal.pgen.1002974 (PMC3459985; doi:10.1371/journal.pgen.1002974)

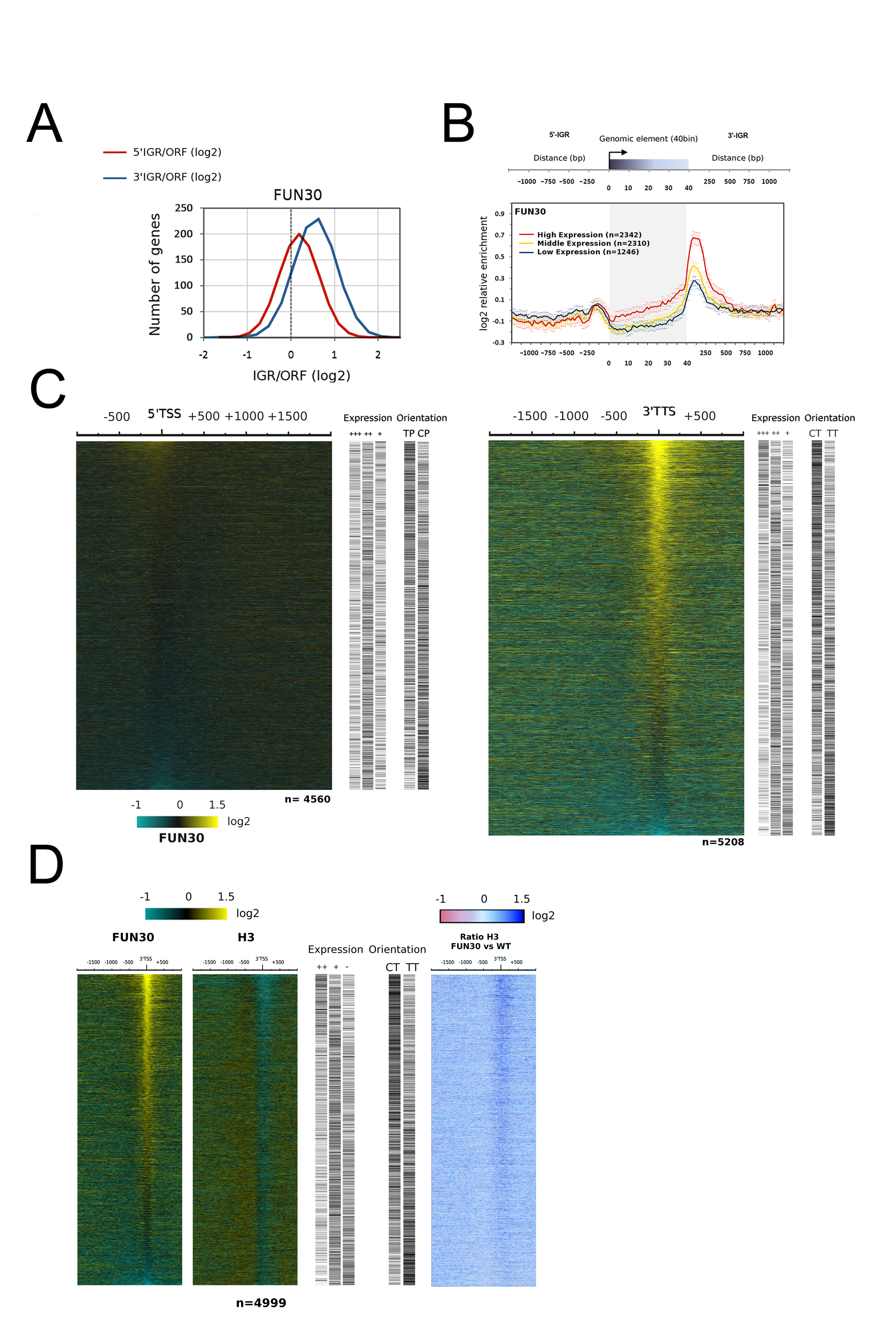

Supplement: Figure S1 — Fun30 preferentially binds intergenic regions over coding regions (ORFs). A) Analysis of Fun30 binding to intergenic regions, red line: ratio of 5′ intergenic versus ORF regions, blue line: ratio 3′ intergenic versus ORF regions. B) Fun30 shows a pronounced binding at the intergenic 3′ end region and this enrichment is directly correlated with expression levels, shown is the average gene analysis for Fun30 binding in relation to RNA transcript levels (determined by RNA-seq in this study) number of genes (n) in each category is indicated in the figure, error bars: 95% confidence interval. C) left panel: Binding profile of Fun30 relative to 5 Transcriptional Start Sites (5′TSS). The clusters contain 4560 genes where the 5′TSS has been identified [89]. Grey bars on right side indicate respectively expression level for each gene and promoter orientation (TP: Tandem Promoters, CP: Convergent Promoters). Values are represented in log2; right panel: Binding profile of Fun30 relative to 3′ Transcriptional Termination Sites (3′TTS). The clusters contain 5208 genes with identified 3′TTS [89]. Grey bars on right side indicate respectively expression level for each gene and promoter orientation (CT: Convergent Terminators, TT: Tandem Terminators). Values are represented in log2. D) As in (C), right panel, but corresponding histone H3 occupancy in wildtype cells and the change in histone H3 occupancy as ratio of occupancy between fun30-deleted/wildtype cells is shown. (TIF) [file pgen.1002974.s001.tif]

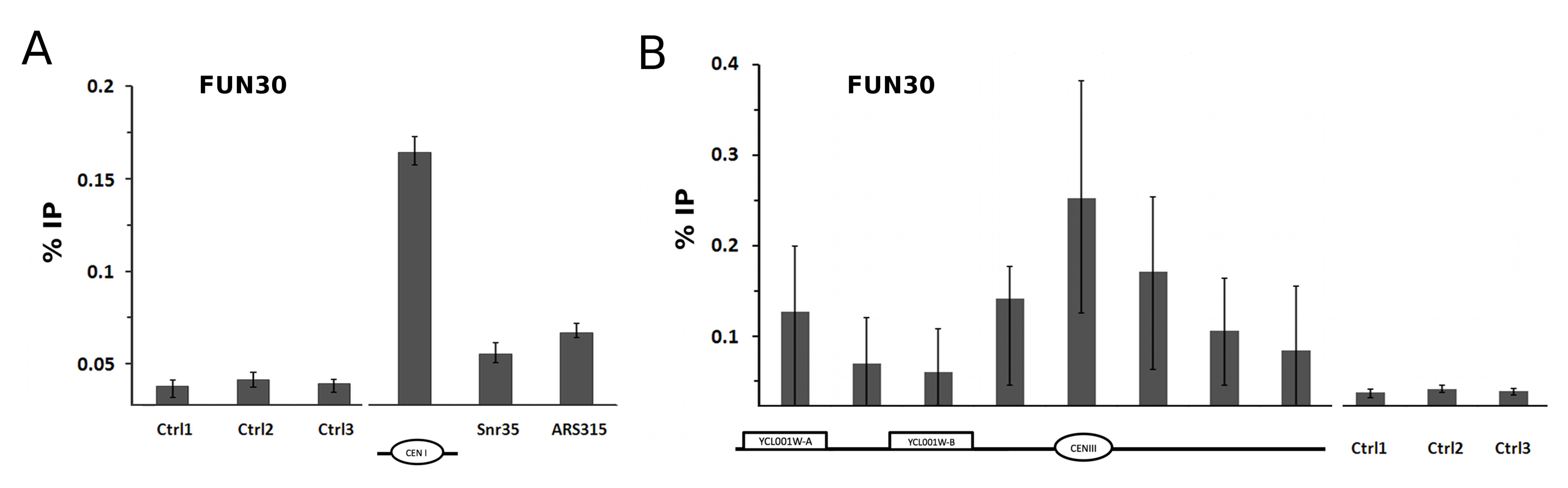

Supplement: Figure S2 — Fun30 is enriched over centromeric regions. A) Validation of Fun30 binding sites by ChIP following qPCR. Fun30 binding at control regions (Ctrl1–3) where Fun30 did not bind according to our ChIP-seq data, and binding to CEN1, snR35, ARS315. B) Fun30 binding to CEN3 and surrounding region using primer pairs spanning +/−2 kb, controls as in (A). Level of enrichment is expressed as % DNA precipitated as compared to input. Background binding to beads was substracted, error bars represent the difference of the maximum and minimum values from the mean. Shown are results from 2 biological replicas, each with 2 technical replicas. (TIF) [file pgen.1002974.s002.tif]

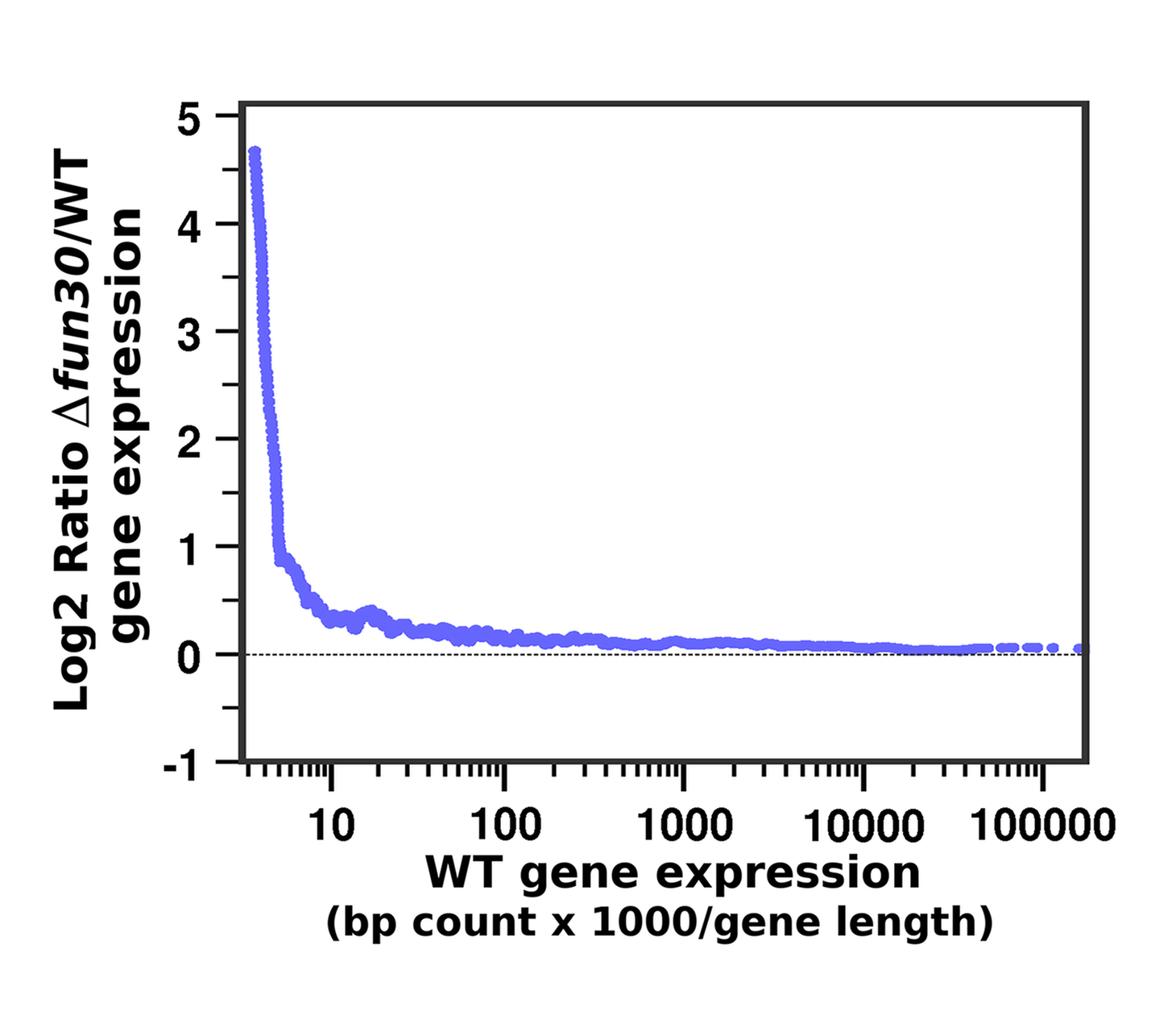

Supplement: Figure S3 — Fun30 primarily acts as a repressor of transcription. Moving average plot (window size = 150 genes, step size = 1 gene) of the mRNA transcription level ratios in Y00389 (Δfun30) versus BY4741/Y00000 (WT) plotted as a function of mRNA levels in WT at 30°C in YPD (normalized reads intensity by bases pairs count ×1000/divided by gene length, [27]). The gene expression ratio from Δfun30 versus wildtype was plotted as moving average of this ratio as a function of the wildtype gene expression levels. The global expression profile for the Δfun30 mutant showed a ratio of 1 for moderately expressed and highly expressed genes indicating that Fun30 does not affect genes at this range of expression levels. Silent or weakly expressed genes in wildtype showed a high median expression ratio. (TIF) [file pgen.1002974.s003.tif]

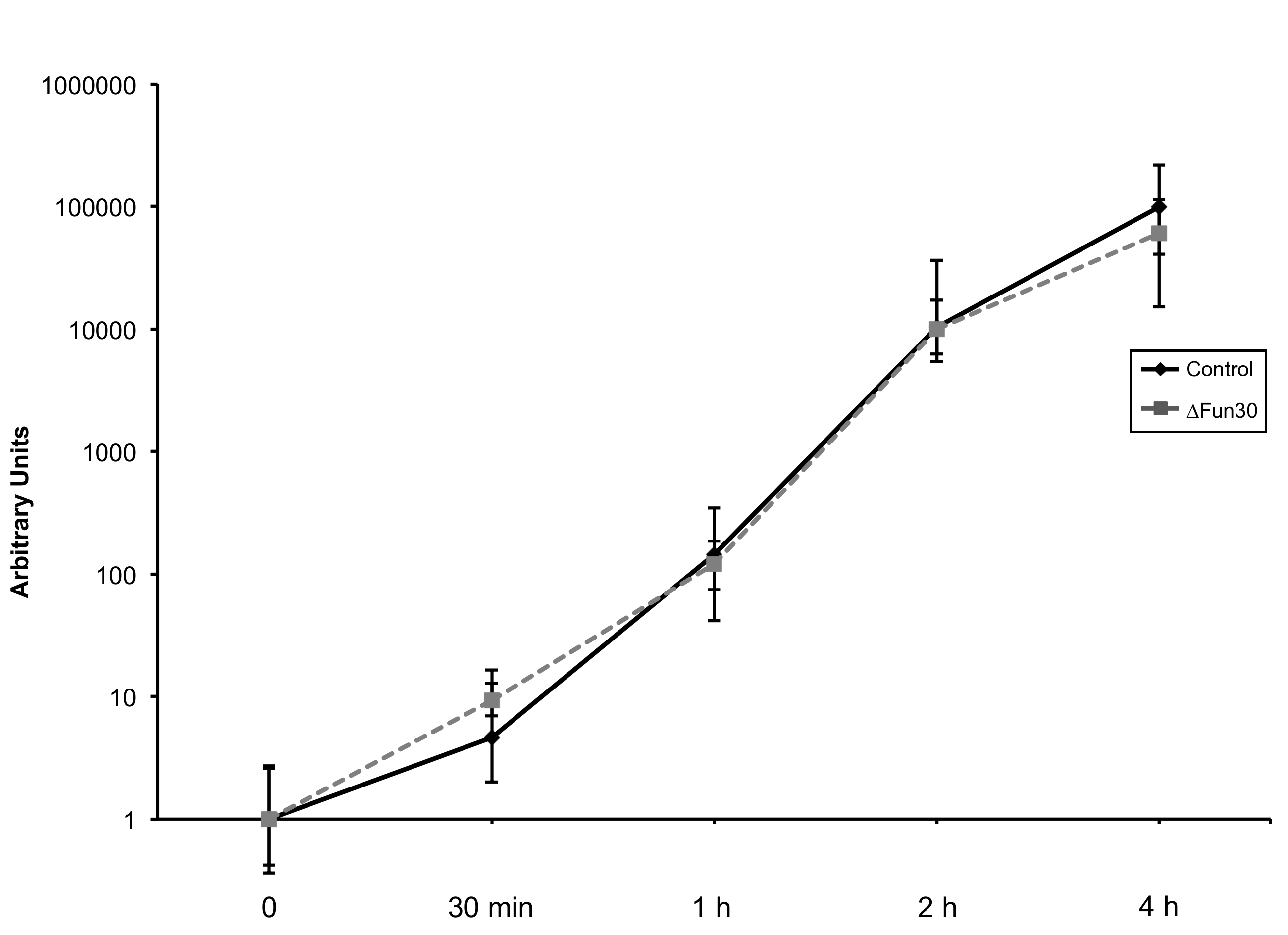

Supplement: Figure S4 — Deletion of Fun30 does not affect promoter activity of GAL1 integrated at centromere CEN3. Analysis of GAL1 promoter induction at CEN3 locus. RNA from SLY806 (control, black) and SC117 (Δfun30, dashed gray) strains was isolated at indicated time points after addition of galactose and analyzed by RT-qPCR using primers specific for CEN3 locus (PM22/PM48). (TIF) [file pgen.1002974.s004.tif]

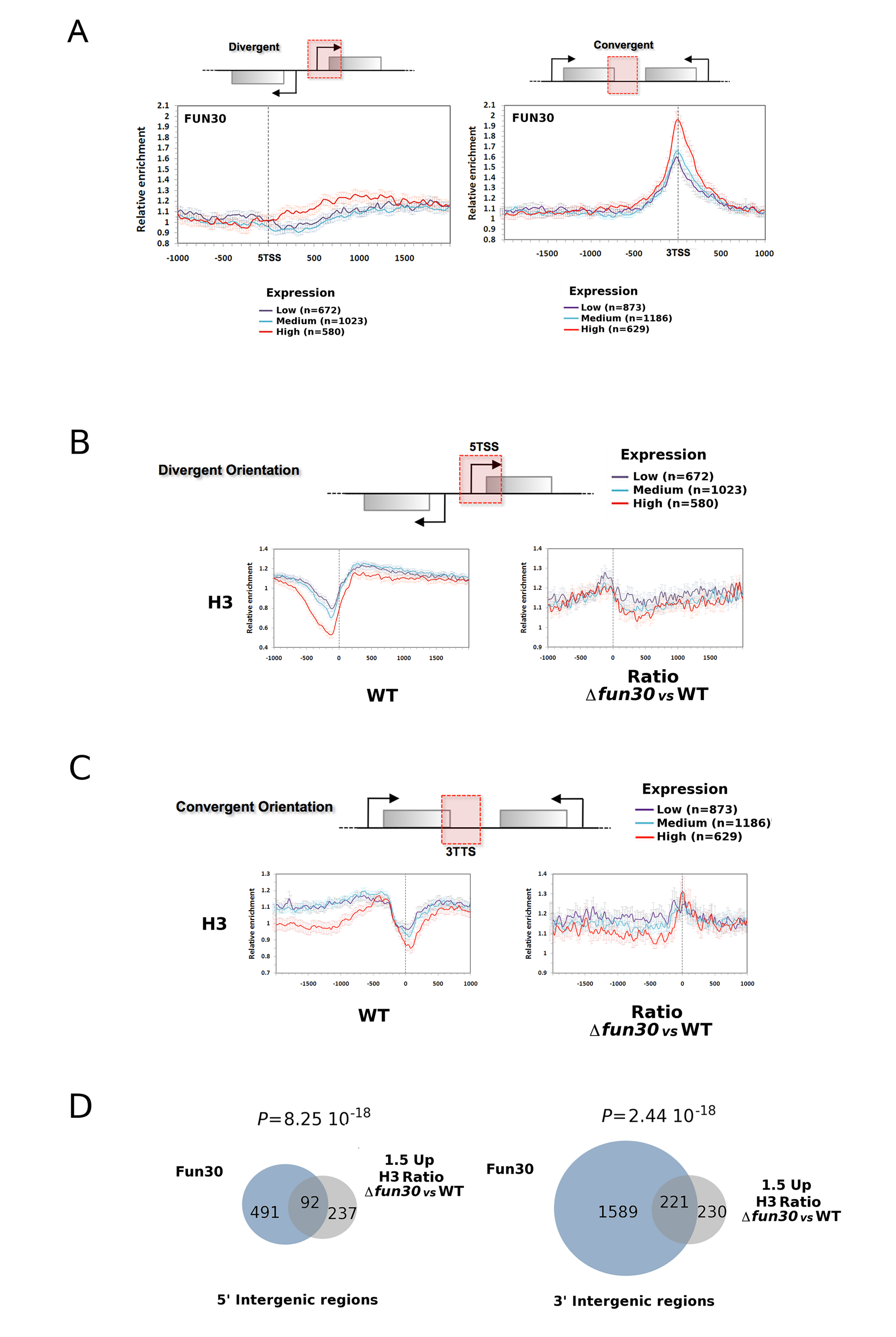

Supplement: Figure S5 — Fun30 regulates histone H3 occupancy at intergenic regions. A) 3′ regions of genes show the greatest enrichment for Fun30 when compared to promoter or coding regions. Average occupancy of Fun30 for divergent orientation promoters (left panel) and convergent terminators (right panel). The data were binned into three groups (High, medium, low) according to the expression level of wildtype cells. The number of genes (n) in each category is indicated. Error bars represent 95% confidence intervals. The genomic region of interest was divided into 40 equally sized bins. The 5′ and 3′ flanking regions have 1250 bp from respectively the beginning and the end site of the genomic elements and divided into a 50 fragments of 50 bases (see Materials and Methods for full description). (B, C) Average trends in specific promoter or terminator regions - i.e. only divergent or convergent genes - were determined for the histone H3 occupancy profiles in wildtype and Δfun30 cells. As previously shown, histone H3 is mainly present within coding regions whereas in both promoter and terminator specific regions histone H3 is relatively depleted. In Δfun30 mutants there is an increase of histone H3 at promoter and terminator regions. B) Average occupancy analysis for histone H3 for divergent orientation genes relative to the 5′TSS position for wildtype cells (left panel) and ratio Δfun30 versus WT (right panel, Y00389 versus BY4741/Y00000). C) Average occupancy analysis for histone H3 for convergent orientation genes relative to the 3′TTS position of genes for wildtype cells (left panel) and ratio Δfun30 versus WT (right panel, Y00389 versus BY4741/Y00000). D) Venn diagrams showing results of hypergeometric probability tests for 5′IGR and 3′IGR Fun30 targets and changes in histone H3 in Δfun30 mutant. This analysis revealed that the changes in histone H3 occupancy are significant for the 5′ and 3′ intergenic regions. Venn diagram illustrating the overlaps in 5′ intergenic regions (5′IGR) and [file pgen.1002974.s005.tif]

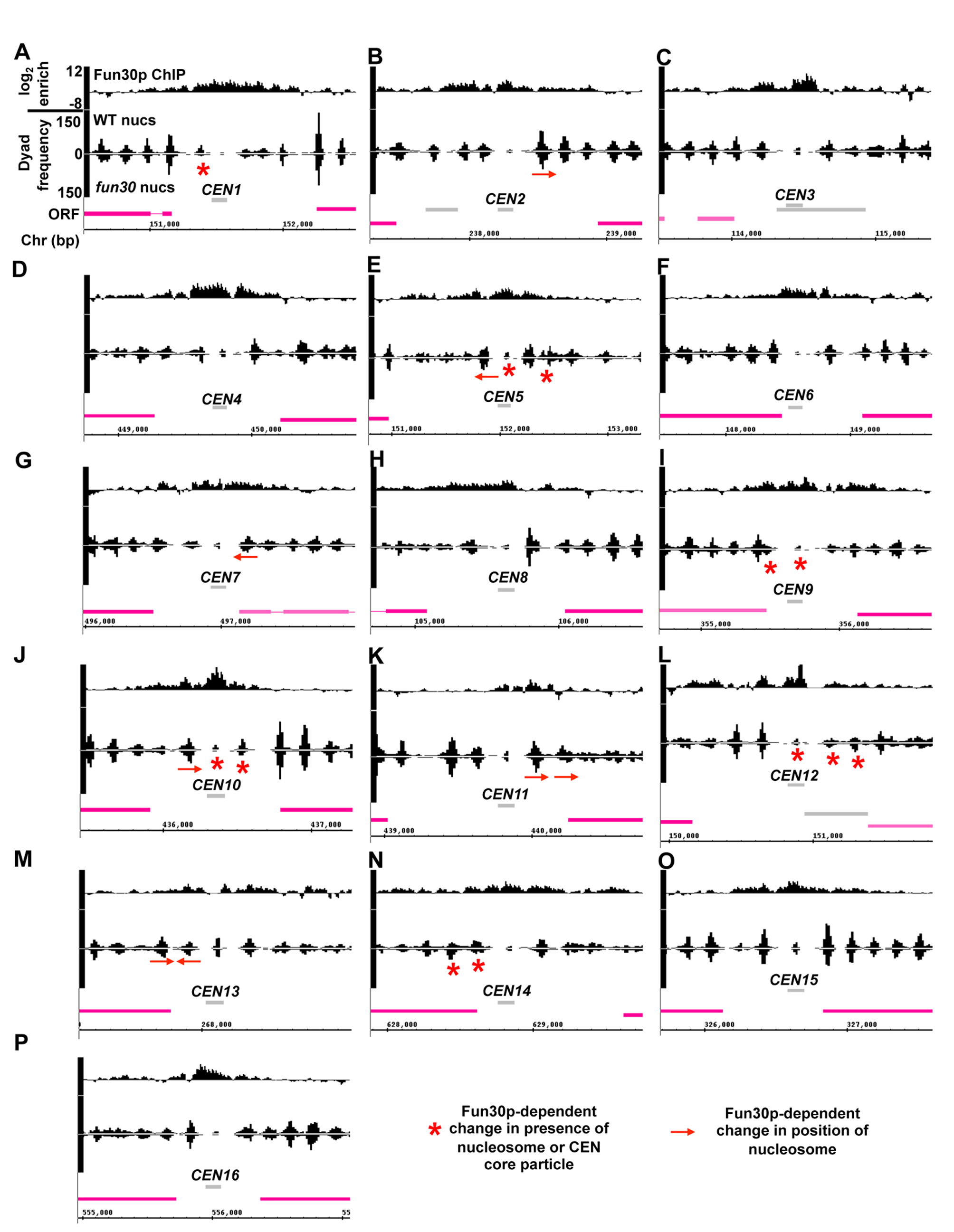

Supplement: Figure S6 — The majority of yeast CENs exhibits Fun30-dependent changes in flanking nucleosome position and/or CEN core MNase accessibility. A) Genome browser traces of Fun30 ChIP enrichment and nucleosome dyad frequency centered on and surrounding yeast CEN1; B) CEN2; C) CEN3; D) CEN4; E) CEN5; F) CEN6; G) CEN7; H) CEN8; I) CEN9; J) CEN10; K) CEN11; L) CEN12; M) CEN13; N) CEN14; O) CEN15; P) CEN16. The upper trace of each panel shows log2 Fun30 ChIP enrichment values binned at 10 bp intervals and smoothed with a 3 bin moving average. The wild-type (WT) and Δfun30 mutant nucleosome (nuc) traces were plotted as mirror images in the lower panel. Centre point positions of paired sequence reads with end-to-end distances of 150 bp+/−20% were mapped across the yeast genome for the wild-type and Δfun30 mutant MNase-digested chromatin sequencing samples, binned at 10 bp intervals, and the resulting frequency distributions smoothed by applying a 3 bin moving average. This class of size-selected paired-end sequence reads largely defines the DNA entry- and exit-points on nucleosomes exposed by MNase digestion in the original chromatin sample. The frequency distributions of paired-read center points therefore effectively estimates the frequency of nucleosome dyads [54], [55] and peaks in the distribution correspond to translationally positioned nucleosomes in the original genome. The CEN core particle is also mapped using this method and can be visualized as a small peak centered on the CEN region marked with a grey box on each browser panel, pink boxes mark the surrounding ORFs. Fun30-dependent changes in the height of a nucleosome dyad or CEN core particle peak are marked with a red asterix. Fun30-dependent changes in the position of a CEN-flanking nucleosome dyad peak are marked with red arrows. (TIF) [file pgen.1002974.s006.tif]

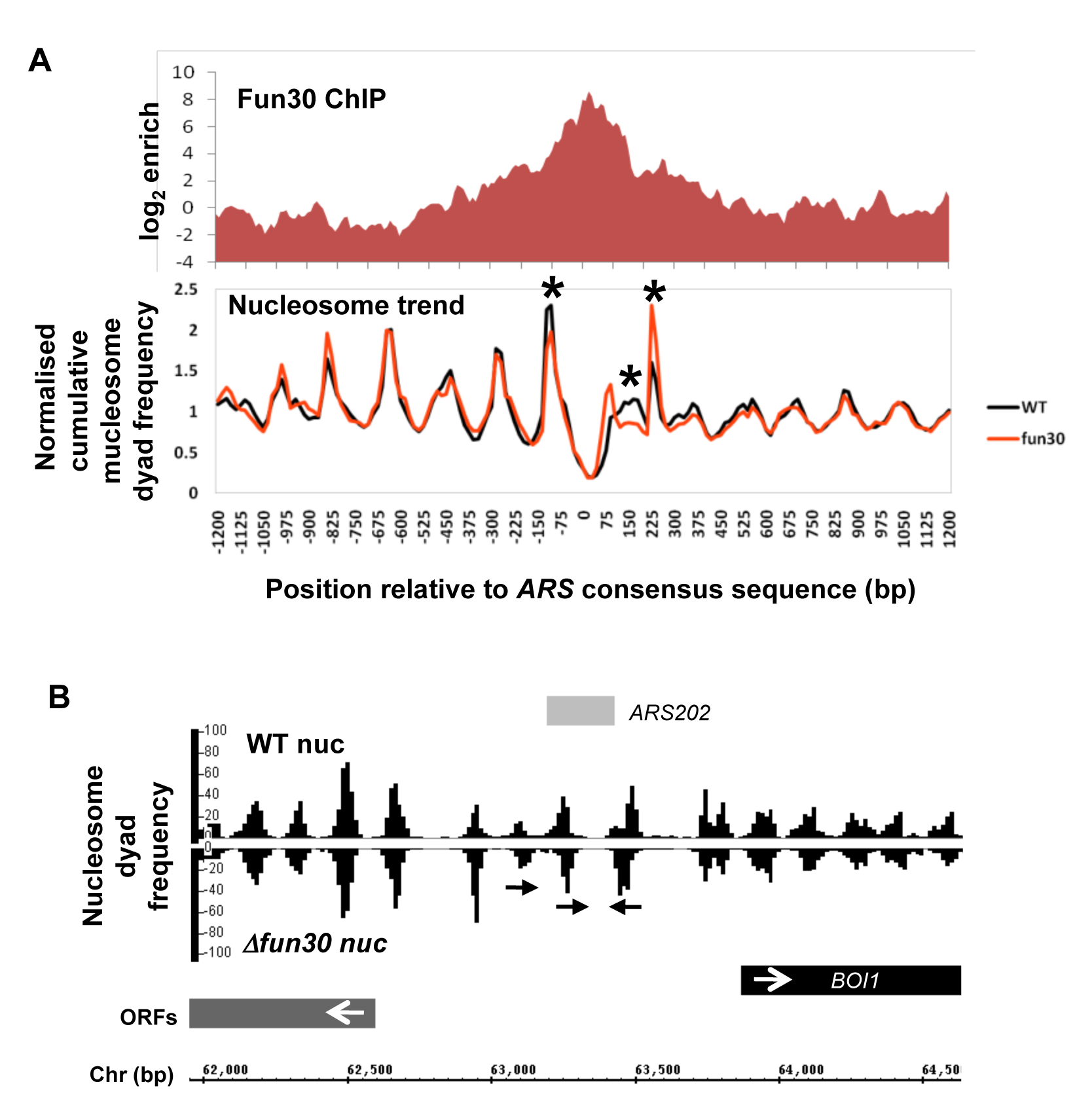

Supplement: Figure S7 — Fun30 is required for normal nucleosome positioning at other sites identified by ChIP-seq. A. ARS regions show Fun30-dependent nucleosome positioning surrounding the ARS consensus sequence (ACS). The upper graph shows a plot of the cumulative Log2 Fun30 ChIP enrichment values centered on the yeast ACS elements defined by Nieduszynski et al. [96]. The lower graph shows plots of the cumulative nucleosome dyad frequencies in the same region for wildtype (black line) and Δfun30 mutant (red line) chromatin sequencing data sets. The cumulative nucleosome dyad frequencies in each 15 bp bin were normalized by dividing by the average nucleosome dyad frequency for the whole feature window in order to place “random” nucleosome occupancy at a value of 1. Changes in cumulative distribution between wildtype and the Δfun30 mutant are indicated with asterisks. B. Genome browser trace of nucleosome dyad frequencies at Fun30-dependent ARS202 plotted as described for CEN analyses, Figure 8, Figure S6. Three nucleosomes surrounding the ARS which exhibit Fun30-dependent changes in position are marked with arrows. (TIF) [file pgen.1002974.s007.tif]

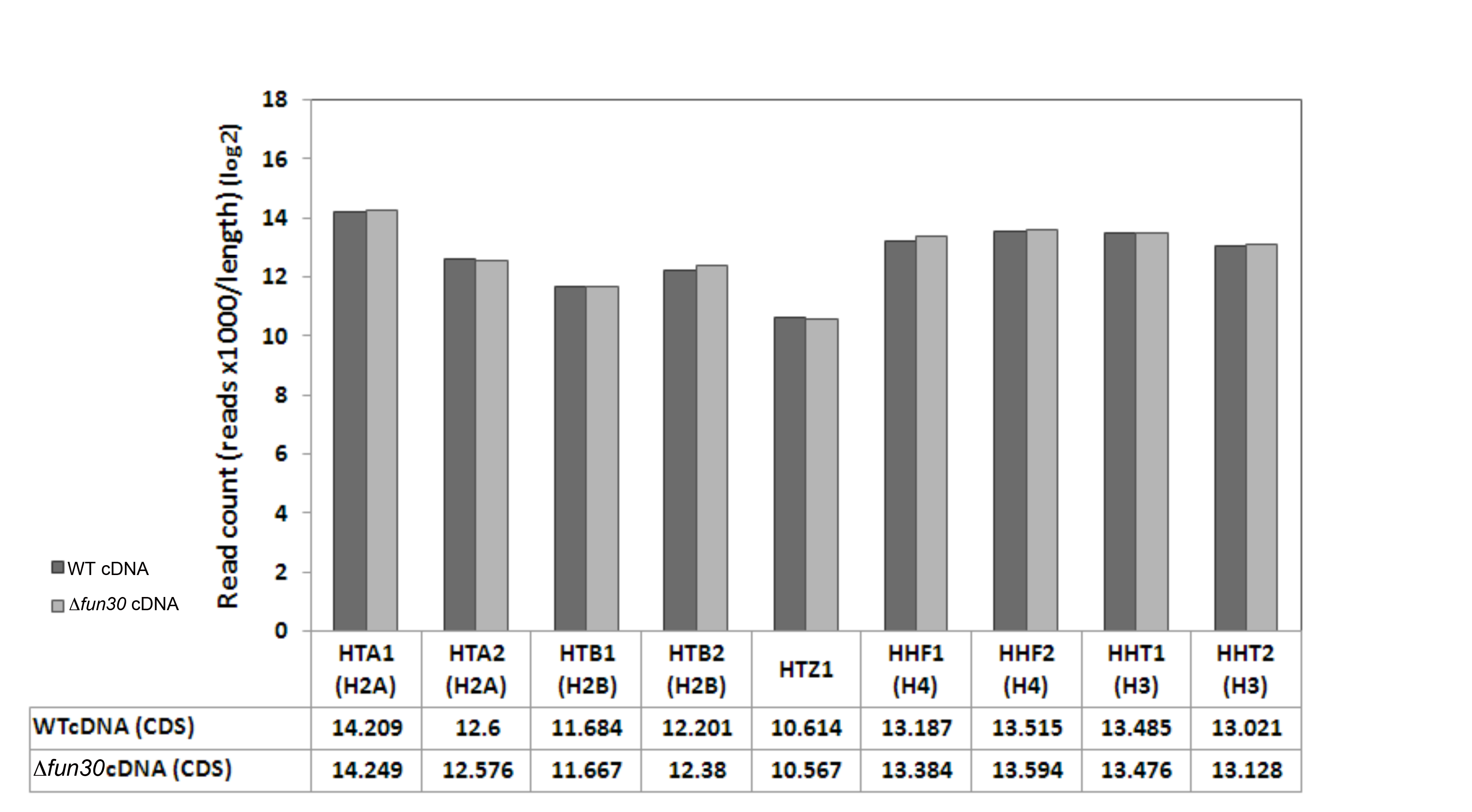

Supplement: Figure S8 — RNA-seq analysis shows that histone gene expression is not changed upon FUN30 deletion. Expression in wildtype cells: dark grey bars, in Δfun30 cells: light grey. (TIF) [file pgen.1002974.s008.tif]

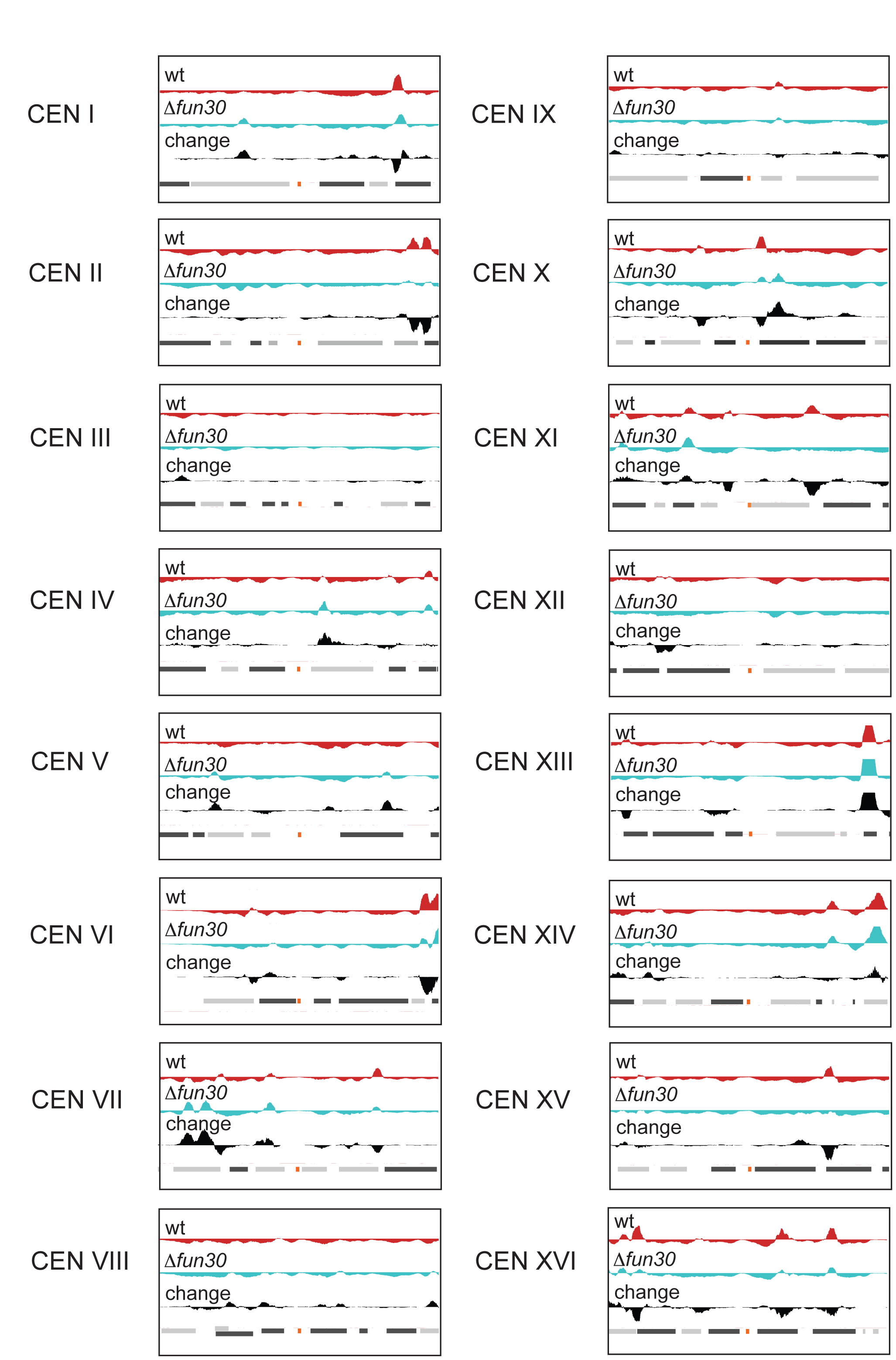

Supplement: Figure S9 — Fun30 affects Htz1 occupancy around a majority of centromeres. Effect of Fun30 on Htz1 occupancy 5 kb up- and downstream of CEN1–16. Shown is Htz1 occupancy from wildtype (wt, red) and Δfun30 cells (light blue, below) expressed as normalized sequence tag counts corrected for input in linear scale. The change in occupancy of Htz1 is indicated in the lane below as the values from the Δfun30 cells minus the values from wt cells (black). Positions of ORFs and centromeres are indicated in the lowest lane, orange box: centromere, back and grey boxes: ORFs in the sense and antisense direction, respectively. Axis and scales as in Figure 6. (TIF) [file pgen.1002974.s009.tif]

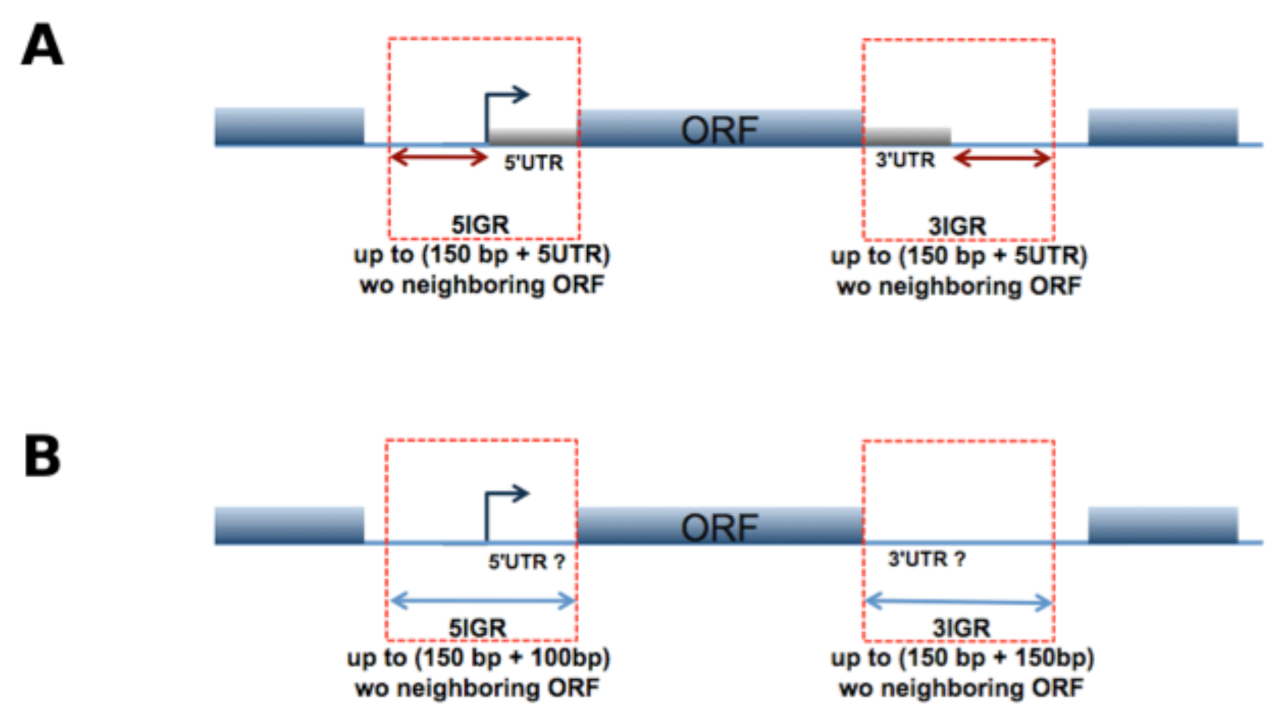

Supplement: Figure S10 — Description of flanking regions annotations for coding gene. A) 5′IGR and 3′IGR region assignment for genes having identified 5′UTR and/or 3′UTR by [89]. B) 5′IGR and 3′IGR region assignment for gene having unidentified 5′UTR and/or 3′UTR. (TIF) [file pgen.1002974.s010.tif]

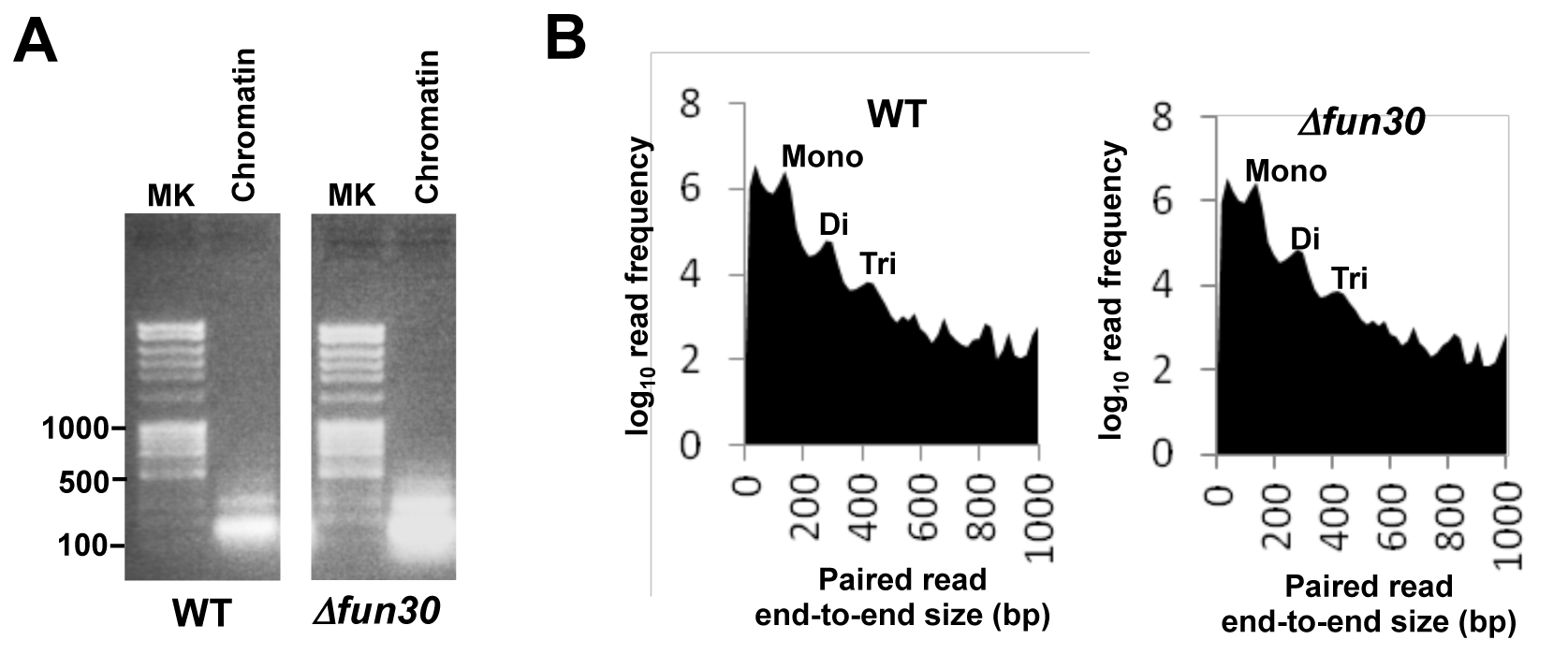

Supplement: Figure S11 — MNase digested chromatin samples processed for paired-end mode Illumina DNA sequencing. A. DNA from MNase digested chromatin fractions purified from wild-type and Δfun30 yeast strains separated by agarose gel electrophoresis and stained with ethidium bromide. B. Graph of the number of aligned paired-end reads obtained by Illumina GAIIx sequencing of material shown in Fig. 1A versus paired-read end-to-end distance (SAM format ISIZE value). Peaks at ∼150 bp, 300 bp and 450 bp are marked and correspond to mono-, di- and tri-nucleosome DNA fractions respectively. The end-to-end distances of paired sequence reads therefore reflect the distribution of chromatin particle input DNA. (TIF) [file pgen.1002974.s011.tif]
